# Automated and parallelized spike collision tests to identify spike signal projections

**DOI:** 10.1016/j.isci.2022.105071

**Published:** 2022-09-05

**Authors:** Keita Mitani, Masanori Kawabata, Yoshikazu Isomura, Yutaka Sakai

**Affiliations:** 1 Brain Science Institute, Tamagawa University, Machida, Tokyo, Japan; 2 Department of Physiology and Cell Biology, Graduate School of Medical and Dental Sciences, Tokyo Medical and Dental University, Tokyo, Japan

**Keywords:** Techniques in neuroscience, Biological sciences research methodologies, Control Systems

## Abstract

The spike collision test is a highly reliable technique to identify the axonal projection of a neuron recorded electrophysiologically for investigating functional spike information among brain areas. It is potentially applicable to more neuronal projections by combining multi-channel recording with optogenetic stimulation. Yet, it remains inefficient and laborious because an experimenter must visually select spikes in every channel and manually repeat spike collision tests for each neuron serially. Here, we automated spike collision tests for all channels in parallel (*Multi-Linc* analysis) in a multi-channel real-time processing system. The rat cortical neurons identified with this technique displayed physiological spike features consistent with excitatory projection neurons. Their antidromic spikes were similar in shape but slightly larger in amplitude compared with spontaneous spikes. In addition, we demonstrated simultaneous identification of reciprocal or bifurcating projections among cortical areas. Thus, our *Multi-Linc* analysis will be a powerful approach to elucidate interareal spike communication.

## Highlights

• We identified spike signals of multiple projections among different brain areas
• Our method was realized by the automation and parallelization of spike collision tests
• More than ten projections were simultaneously identified in our demonstration
• Reciprocal or bifurcating interareal projections were simultaneously identified

## Graphical abstract

**Figure:**
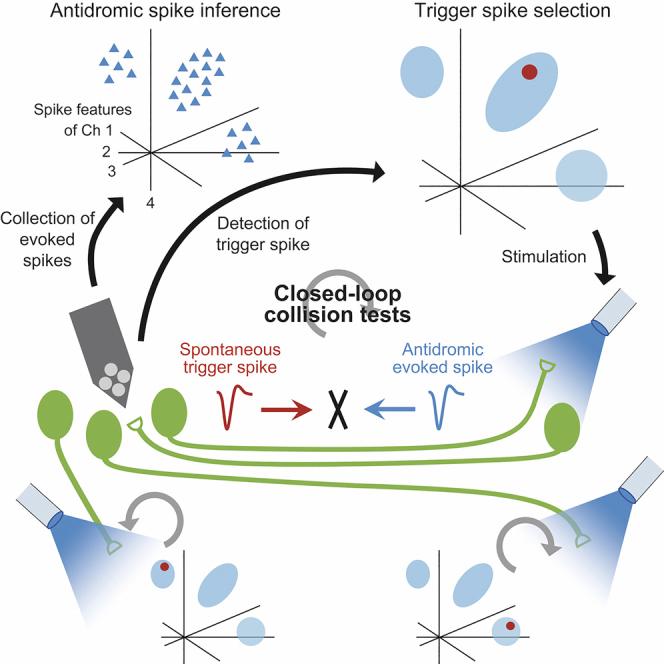

## Introduction

**Figure 1:**
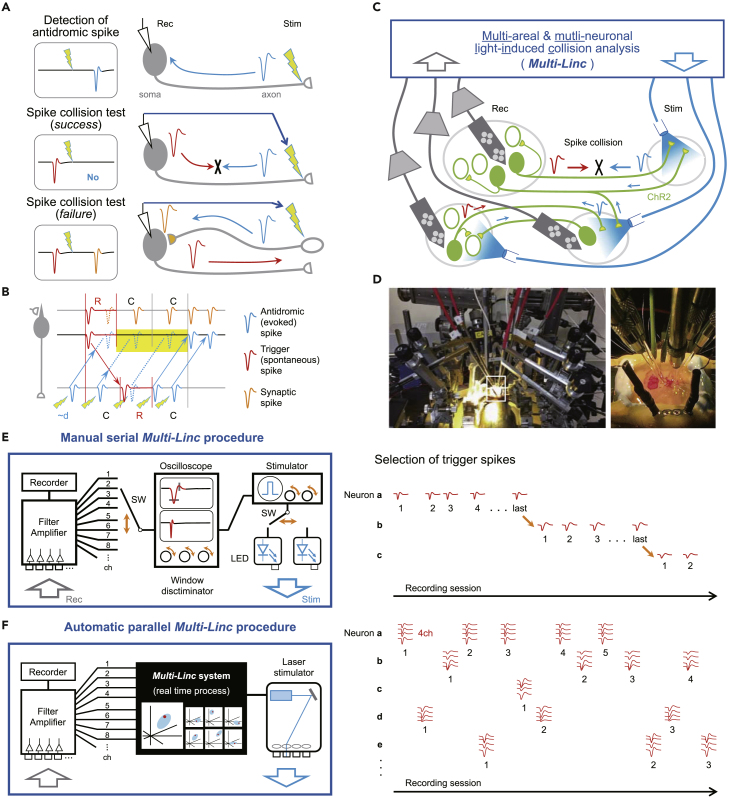
Automation and parallelization of the spike collision test (A) Principle of the spike collision test. An evoked antidromic spike (upper) disappears upon colliding with a spontaneous (trigger) spike on the same axon (middle). Otherwise, a synaptic spike persists (lower). (B) Stimulation timings to cause spike elimination (dashed lines) by a neuronal refractory period, by spike collision, and no elimination (solid line). Spike collision eliminates only antidromic spikes, whereas the refractory period eliminates both antidromic and synaptic spikes. (C) The concept of the *Multi-Linc* analysis. Multi-unit recordings (Rec) are combined with optogenetic stimulation (Stim) to test spike collisions for multiple projection neurons among brain areas. (D) A *Multi-Linc* experiment in a rat (left). Several optical fibers and multi-channel silicon probes were placed in different cortical areas (right). (E) Schematic of manual and serial *Multi-Linc* procedures (left). An experimenter needs to continually operate analog-type devices visually and manually (orange arrows; input channel selector, window discriminator, stimulator, LED selector, and so forth) to detect antidromic-like spikes and test spike collisions during the experiment. Consequently, neurons are subject to spike detection and spike collision tests one-by-one serially (right). See Saiki et al. (2018). (F) Schematic of the automatic and parallel *Multi-Linc* procedure (left). A multi-channel *Multi-Linc* system automatically conducts real-time processes to isolate trigger spikes precisely using multi-dimensional spike features (every tetrode from as many as 128 channel inputs) and to select an optimal stimulus to test spike collision within several milliseconds. Many neurons can be efficiently subjected to spike collision tests in parallel (right).

The brain performs various functions by conveying spike signals of individual neurons cooperatively among brain areas. To elucidate such interareal spike communication, it is essential to examine the spike activity of a projection neuron that is proven to send its axon to specific target areas. Extracellular spike (unit) recording is currently the only method that precisely captures every spike in any brain area of a behaving animal. The spike collision test can reliably determine the axonal projection of an extracellularly recorded neuron without requiring visualization (Bishop et al., 1962; Lipski, 1981). This test is based on the principle that two spikes always disappear if they collide with each other on the same axon. When we stimulate the axon in the target area, an evoked “antidromic” spike will be detected at the soma with a delay (Figure 1A, upper; typically a few to tens of milliseconds later). Then, if the antidromic spike is evoked immediately after a spontaneous (“trigger”) spike is detected at the soma, they will collide and disappear on the axon and the antidromic spike will not be detected (Figure 1A, middle). In contrast, if the recorded neuron is excited by other activated neurons via synapses, an evoked “synaptic” spike will be detected despite the trigger spike (Figure 1A, lower). On the basis of the “success” of spike collision, the target area of the extracellularly recorded neuron can be identified. In the collision test, we should care cases where the evoked spike may be eliminated in the refractory period of the trigger spike, because the evoked synaptic spike can also be eliminated (Figure 1B; Bishop et al., 1962; Fuller and Schlag, 1976; Lipski, 1981). To avoid false positive identification, the latency of antidromic spikes should be sufficiently longer than the refractory period of the neuron (*L* = *d* + *C* > *R*, where *L* is the latency consisting of the evoking delay *d* and the conduction time through the axon *C*, and *R* is the refractory period). Spike collision can be judged when the axon could be stimulated in the range from *d* + *C − R* to *C* + *R − d* after the trigger spike.

For the past 50 years, this analysis has been used to identify projection neurons in various brain areas, leading to fruitful findings on neural circuitry (e.g., Evarts, 1968; Wilson, 1987; Turner and DeLong, 2000; Everling and Munoz, 2000). However, this classical analysis is extremely difficult and inefficient for technical reasons. First, it is laborious to search for a single neuron with evoked spikes by advancing a recording electrode little by little. Second, electrical stimulation generates a huge electrical artifact that obstructs spike detection. Third, the stimulation is prone to make an electrolytic lesion or insensitivity near the tip of the electrode.

Recently, several laboratories have employed multi-channel recording and optogenetic stimulation for the spike collision test to solve the aforementioned problems (Jennings et al., 2013; Li et al., 2015; Economo et al., 2018; Saiki et al., 2018). For example, we proposed a concept of “multi-areal and multi-neuronal light-induced collision” (*Multi-Linc*) analysis, which repeats to test spike collisions for axonal projections of multiple neurons from multiple areas to other areas all at once (Figure 1C; Saiki et al., 2018). To prioritize the efficiency of *Multi-Linc* analysis, as many spike collisions as possible are tested even though they are tentative and inaccurate *online* at the experimental stage; they are later re-evaluated more accurately to judge success *offline* at the analytical stage. This analysis has been used to identify distinct types of cortical (Soma et al., 2017; Saiki et al., 2018; Rios et al., 2019; Hamada et al., 2021) and striatal (Nonomura et al., 2018) projection neurons by combining multi-unit recording through tetrodes with optogenetic stimulation in behaving rats expressing Channelrhodopsin-2 (ChR2) (Figure 1D).

In the original *Multi-Linc* procedure, an experimenter selects one recording channel and one stimulation site manually and searches the trigger spike matched with one of the evoked spikes on an oscilloscope visually and manually (Figure 1E, left, orange arrows; Saiki et al., 2018). Consequently, the experimenter must wait for tens of spike collision trials to complete in a single neuron before searching for the next neuron (Figure 1E, right). Such manual series operations and visual spike isolation seriously degrade the efficiency and accuracy, respectively, of multi-channel spike collision tests. If trigger spikes are precisely selected in a multi-dimensional space of spike features for each tetrode automatically within several milliseconds (Figure 1F, left), spike collision tests can be conducted in parallel for multiple neurons (Figure 1F, right). Thus, the automation and parallelization of the *Multi-Linc* procedure are expected to improve the efficiency and accuracy of spike collision experiments. The key technical issues to be solved are 1) automatic detection of inferred antidromic spikes from noisy background signals, 2) real-time selection of trigger spikes for spike collision tests, and 3) implementation of reliable real-time processing (on a millisecond timescale) in a computer system with multi-channel inputs and outputs.

In the present work, we have overcome these technical issues in both hardware and software and have established an automatic and parallel *Multi-Linc* system that is real-time computerized for multi-channel spike collision tests. Using this novel system, we succeeded in automatically identifying cortical projection neurons recorded in the motor cortex of ChR2-expressing transgenic rats. As far as we know, this study is the first to report the automation and parallelization of spike collision tests for multiple neurons. We expect our *Multi-Linc* analysis method to provide insights into the principle of fast spike communication among brain areas in the future.

## Results

### Purposes of online and offline procedures with two protocols

**Figure 2:**
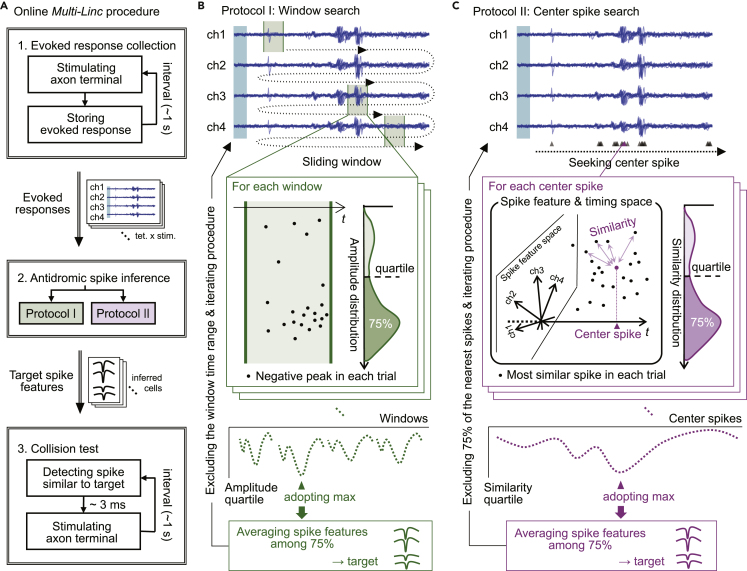
Automatic and parallel *Multi-Linc* procedure (A) Flow of the online *Multi-Linc* procedure. (B and C) Two protocols to infer antidromic spikes of an identical neuron among multiple evoked spikes. The schema corresponds to a procedure on a certain combination of a tetrode and a stimulation site. (B) Window search protocol (protocol I). Evaluation of evoked negative peaks within a sliding time window determines candidate antidromic spikes. (C) Center spike search protocol (protocol II). All evoked spikes in every stimulation trial are sought. The similarity of each spike to the most similar spike in every other trial is evaluated. “Similarity” is defined in a space of spike features and timing. Typically, the peak pattern of a spike in four channels of a tetrode can form a space of spike features. Evaluation of aggregation in a high-dimensional space of the evoked spike feature and timing determines the candidate antidromic spikes.

Our *Multi-Linc* analysis consisted of the online (real-time) control of experiments to collect data (Figure 2) and the offline judgment to identify neuronal projections from the collected data (Figure S1). Although common procedures were used in parts of the online control and the offline judgment, the purposes are different. The purpose of the online controller is to control closed-loop stimulations to efficiently collect results of spike collision tests. The purpose of the offline judgment is to validate the identification of neuronal projections from the whole collected data.

For the common part of the online and offline procedures, we attempted two different protocols (protocols I and II) because they enabled cross-validation with each other such that a dataset collected with protocol I (online) was validated with protocol II (offline), and vice versa. Multiple validations were important because there was no previous benchmark for automated spike collision test.

### Procedures in online controller

#### Overview of online controller

First, we briefly describe procedures in the online controller. We composed the automatic and parallel *Multi-Linc* procedure of three serial steps for online control of experiments (Figure 2A; see STAR Methods for details): collection of evoked responses, inference of antidromic spikes in evoked responses, and spike collision test for inferred antidromic spikes. The first two steps correspond to the antidromic spike detection in Figure 1A, top, and the third corresponds to the spike collision test in Figure 1A, middle and bottom. In the evoked response collection (Figure 2A, first block), one of the candidate projection sites in which optical fibers are placed is selected and stimulated optogenetically at an arbitrary timing. Simultaneous recordings through multi-tetrode probes inserted into multiple brain areas (Saiki et al., 2018; Kawabata et al., 2020) are stored for several tens of milliseconds after the stimulation (evoked responses). The slow component of the change in potential is removed by a high-pass filter such that only the spike activity remains. The next stimulation site and timing are selected after a certain interval (∼1 s) to avoid residual effects of artificially evoked neural activity. The evoked response collection (first block in Figure 2A) is completed after a sufficient number of stimulations per stimulation site (typically 100 times per site). The set of evoked responses is then obtained for every combination of tetrodes and stimulation sites, which is delivered to the next step (second block in Figure 2A).

If a neuron around tetrode projects to a stimulation site, antidromic spikes might be observed in the evoked responses. However, the evoked responses typically contain many synaptic spikes through the neuronal population. Occasionally, antidromic spikes of different neurons might be mixed. The purpose of the antidromic spike inference (second block in Figure 2A) is to sort a group of spikes likely to be antidromic spikes of an identical neuron from contaminated multiple spikes in the noisy evoked responses. If synaptic spikes or antidromic spikes of other neurons contaminate the inferred group of spikes, then incorrect spontaneous spikes are likely to be selected as triggers for spike collision tests, which would decrease the success rate of spike collisions. Therefore, the accuracy of antidromic spike inference is extremely important in the online *Multi-Linc* procedure. Antidromic spikes should be observed with stable latency from stimulation timings because the spike conduction time should be determined by the excitability and length of the axon. By contrast, synaptic spikes might be evoked at various timings depending on the states of the neuronal population. The problem lies in the difficulty associated with segregating temporally aggregated spikes from noisy timing spikes. To solve this problem, we adopted two types of protocols: “window search” (protocol I; Figure 2A, second block, green) and “center spike search” (protocol II; magenta). These two protocols were also used in offline judgment of spike collision after experiments (Figure S1A).

#### Protocol I for antidromic spike inference

Protocol I simply automated manual procedures of experimenters in classical collision tests (e.g., Saiki et al., 2018). Temporal aggregation is evaluated using a short temporal window independently for the four channels of a tetrode. For each combination of a tetrode and a stimulation site, the temporal window is slid a long time in the filtered evoked responses from channels 1 to 4 (Figure 2B, top). A spike waveform exhibits a sharp negative peak in extracellular recording. Thus, stably large negative peaks within a short temporal window indicate temporally aggregated spikes. For each window, negative peaks (dots in Figure 2B, middle left) are determined in respective trials of stimulations and the distribution of peak amplitudes is obtained (Figure 2B, middle right). The goodness of the window can be characterized by the first quartile of the amplitude (75% from the largest) in the amplitude distribution because a larger amplitude quartile indicates a more stable occurrence of spikes within the window. All the windows of timings and channels are characterized in the same manner. The windows with amplitude quartiles below a certain threshold are excluded from candidates. Among the remaining candidates, the window with the maximum amplitude quartile is adopted as the best window (Figure 2B, bottom). Here, we used the negative peaks with an amplitude greater than the amplitude quartile (75% from the largest) within the adopted window as typical spike waveforms likely to be antidromic spikes of an identical neuron. The spike waveforms corresponding to 75% of negative peaks are averaged for the representative waveform of inferred antidromic spikes. Although the adopted window is defined in a certain channel of the tetrode, the waveform patterns for all four channels are averaged. The feature of the averaged waveform pattern is then delivered to the next step (Figure 2A, third block) as the target of the spike collision test. The windows overlapping the time range of the adopted window are excluded from candidates; the selection of the best window among the remaining candidates is then iterated.

#### Protocol II for antidromic spike inference

Protocol II utilized multi-channel information that is difficult for experimenters to catch simultaneously. Not only temporal aggregation but also the aggregation of spike waveform features of multi-channels is evaluated on the basis of the similarity between spikes. Spike timings are detected within the evoked responses for all trials of stimulations (triangles in Figure 2C, top). On the basis of timings and waveform features of the detected spikes, the aggregation of spikes is searched, and the center spike of the aggregation is determined. For each spike, the nearest spikes in respective trials are determined (dots in Figure 2C, middle). The similarity between spikes is defined in the multi-dimensional space of spike timing and features (see STAR Methods). The degree of aggregation can be characterized by the first quartile in the similarity distribution (75% from the most similar one; Figure 2C, middle right). All detected spikes are characterized by the similarity quartiles in the same manner. Spikes with the similarity quartiles below a certain threshold are excluded from candidates for the center spike. Among the remaining candidates, the center spike with the maximum similarity quartile is adopted as the center of the aggregation (Figure 2C, bottom). In the same manner as protocol I, protocol II uses 75% of the spikes nearest the adopted center spike as typical spike waveforms likely to be antidromic spikes of an identical neuron. The waveform patterns of 75% are averaged for the target of the spike collision test. Once adopted, 75% of the nearest spikes are excluded from candidates, and the selection of the center spike is iterated.

#### Closed-loop control of spike collision tests

In the spike collision test (Figure 2A, third block), spontaneous spikes are detected in real-time and in parallel on all tetrodes. If a detected spontaneous spike is close to either of the target spike features in the same tetrode, then the spike is adopted as a trigger for the collision test. The stimulation site attributed to the target is immediately stimulated. The stimulation should start within a few milliseconds after the occurrence of the trigger spike for the spike collision to be successful. This process requires the highest performance of real-time processing. Acquiring data from multiple tetrodes, filtering all channels, detecting spikes, determining the similarities to target spikes, and exciting a laser requires time. We implemented algorithms for easy filtering and spike detection and selected hardware components to achieve stimulation within 3.2 ms, acquiring data from 32 tetrodes (128 channels).

### Experimental data collection

We conducted experiments using the online *Multi-Linc* controller (Figure 2A) by implementing either protocol I or II in the bilateral motor cortices of ChR2-expressing transgenic rats. In this experiment, two 32-channel silicon probes were inserted bilaterally or unilaterally into the motor cortex of an awake head-fixed rat, and up to six optical fibers were placed on the surface of cortical areas receiving direct projection from the moto cortex anatomically (Figure 1D). The average duration of sessions was 89.0 ± 50.2 min in our demonstration experiments. Both protocols worked well and we collected a number of sessions of data (Table S1).

### Procedures of offline judgment

#### Overview of offline judgment

Here, we briefly describe procedures of the offline judgment of projection identification after the experiments (Figures S1 and S2; see STAR Methods). To validate our *Multi-Linc* results, we cross-checked the data by using the two protocols. Namely, we judged offline using both protocols I and II, irrespective of the protocol used in the online controller. In the first step, we inferred antidromic spikes in the whole stimulation trials offline using both protocols I and II, and detected and sorted spikes on each tetrode into clusters of putative identical neurons throughout the experimental session (Figure S1A). We used a standard method of spike sorting in multi-unit recordings (Takekawa et al., 2012). Hereafter, we simply call each spike cluster as “neuron.”

**Figure 3:**
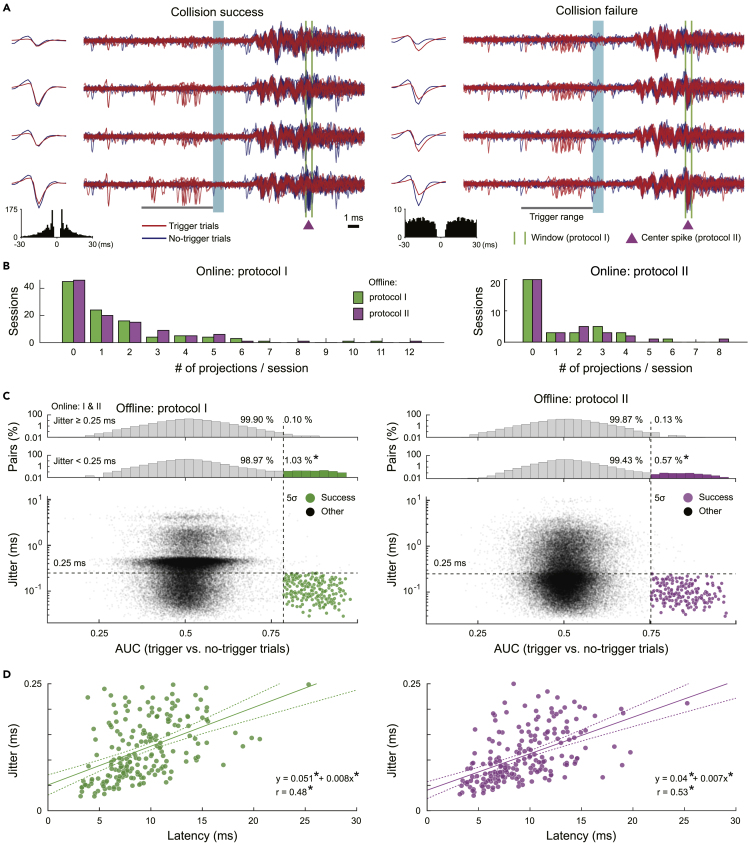
Offline judgment for the identification of neuronal projections (A) Examples of successful (left) and unsuccessful (right) pairs for collision tests, which correspond to different example neurons (spike clusters) tested for a common antidromic spike inference by protocol I (the window indicated by green vertical lines) and protocol II (the center spike indicated by a magenta triangle). Traces recorded by a four-channel tetrode around stimulation timings (light blue) in trigger (red) and no-trigger (blue) trials were overlayed. Median waveforms of trigger spontaneous spikes (red) and evoked spikes in no-trigger trials (blue) are plotted on the left sides. The autocorrelogram of spontaneous spikes was shown at the bottom of each. Spontaneous spikes of the successful neuron (left) within the trigger range (gray bar) eliminated inferred antidromic spikes to be evoked (within the green lines, or around the magenta triangle), whereas spikes of the unsuccessful neuron (right) could not eliminate the antidromic spikes. (B) The number of successfully identified neuronal projections per session (left, online controller by protocol I; right, online controller by protocol II). The outcomes of offline judgements by protocols I and II are plotted as the green and magenta bars, respectively. (C) The definitions of offline judgements by protocols I (left) and II (right). The data sessions obtained by online controllers I and II were gathered. Each dot in the scatterplots indicates the area under the receiver operating characteristic curve (AUC) between trigger and no-trigger trials, which was used as a variable to quantify the elimination of evoked spikes and the jitter of evoked spikes in no-trigger trials (the quartile deviation of the latency) in each tested pair. Upper histograms of AUCs are shown on a logarithmic scale separately for ones with jitter greater than and less than 0.25 ms (horizontal dashed lines; asterisks (∗) indicate p < 0.005 in 2 × 2 *χ*^2^ tests). Green and magenta dots indicate tested pairs judged to pass collision tests on the basis of the defined criteria (AUC > 5*σ* and jitter <0.25 ms), and gray dots indicate the other pairs. (D) The distribution of jitters and latencies of antidromic spikes for the identified neuronal projections. The regression lines (solid) and the 95% confidence intervals (dashed curves) are shown. Asterisks (∗) mean p < 0.005 in Pearson’s correlation tests and linear regression tests.

We judged whether each neuron (cluster) might pass the collision tests for the inferred antidromic spikes and found neurons such that the projection targets were successfully identified (Figure 3A). As a result, multiple projections were simultaneously identified in numerous sessions by both online controllers I and II (left and right in Figure 3B, respectively). The numbers of projections successfully identified in offline judgements I and II were equivalent (green and magenta in Figure 3B, respectively). The majority succeeded in both judgements I and II (Figure S3A). Thus, the results were assured by different criteria. If we also accept a projection judged as success in only one of the protocols I and II, then the successful projections exceeded ten per session at maximum in both online controllers I and II (Figure S3B). We showed the evoked traces with antidromic spikes and with spike collisions for all 13 identified projections from 12 neurons (two different projections were identified in one of the neurons) in the maximum example obtained by online controller I (Figure S4). These were the results of the first use of the online *Multi-Linc* controller; the control parameters have not been fine-tuned. Hence, the maximum outcomes showed that fine-tuning on the basis of the properties of successful neurons should enable the stable identification of more than ten neurons per session. Hereafter, we explain the details of the judgements and the properties of the data sessions collected with online controllers I and II.

#### Definitions of trigger and no-trigger trials

Next, we define the trigger and no-trigger trials for judgements of spike collision tests. We once adopted broad inferences of antidromic spikes, including ones with relatively long jitters, for control comparisons. A set of antidromic spikes inferred to originate in an identical neuron was a target of identification. If the inferred antidromic spikes of an identification target are true antidromic spikes of a certain neuron, then the jitter should be relatively short and a spontaneous spike of the neuron immediately before stimulation should eliminate the evoked antidromic spike by collision in the axon (e.g., Figure 3A, left). We searched such pairs of neurons and identification targets among all the pairs. For each pair, we extracted the trigger stimulation trials in which spontaneous spikes were observed in the time range to cause collision (red traces in Figure 3A; see Figure S1B and STAR Methods). If the number of extracted trigger trials was less than 15, then the pair was excluded for analyses. We also extracted no-trigger trials without spikes in the time range to enable collision for control comparison (blue traces in Figure 3A; see Figure S1B). To ensure a fair comparison, we extracted the no-trigger trials only from trials temporally close to the respective trigger trials in a recording time course. We judged the spike collision on the basis of discrimination between the trigger and no-trigger trials. We also quantified the jitter of evoked spike timings with the quartile deviation among the extracted no-trigger trials. The jitter of synaptically evoked spikes is known to be longer than antidromic ones. On the basis of previous studies on antidromic stimulations by optogenetics (Li et al., 2015; Saiki et al., 2018), we regarded the jitters longer than 0.25 ms (above the horizontal dashed lines in Figure 3C) as controls that could feasibly be synaptic ones.

#### Criteria of judgements for spike collision tests

The elimination of an antidromic spike to be evoked in each trial can be judged by the variables used for the antidromic spike inference—specifically, the peak amplitude within the inferred window in protocol I and the similarity to the inferred center spike in protocol II (see Figures 2B, 2C, S1C, S1D, S2E, and S2F). We applied the receiver operating characteristic (ROC) analyses for the amplitude or the similarity to quantify discrimination between trigger and no-trigger trials. Because the majority of the tested pairs of neurons and identification targets should not match, the discrimination would be non-significant in the majority of pairs. Actually, the areas under the curves (AUCs) of the ROC were distributed around 0.5, the chance level (horizontal values of gray dots in Figure 3C). For jitters shorter than 0.25 ms (horizontal dashed lines); however, the AUC distribution exhibited a long right tail (upper histograms). There were significantly more outliers of the AUC greater than five times the SD(AUC > 5*σ*) for the shorter jitters (<0.25 ms) compared with the fraction for longer jitters (>0.25 ms; p = 6.0 × 10^−56^ in protocol I, p = 1.7 × 10^−16^ in protocol II, *χ*^2^ test). These outliers are likely to be pairs such that the spontaneous spikes of the neurons might eliminate the antidromic spikes to be evoked. We then adopted the pair to be succeeded in the collision test such that the AUC was greater than 5*σ* and the jitter was shorter than 0.25 ms (magenta and green dots in Figure 3C). We confirmed the validity of our judgment in the original scale of the peak amplitude and the similarity (Figure S5).

### Properties of identified neuronal projections

#### Latency and jitter

The latencies of successfully identified antidromic spikes were distributed around 10 ms (Figure 3D). The jitters were linearly correlated with the latencies (*r* = 0.48, p = 2.0 × 10^−12^ in protocol I; *r* = 0.53, p = 2.5 × 10^−15^ in protocol II, Pearson’s correlation test). If it is necessary to identify projections with latencies longer than 20 ms, then the criterion for jitters (<0.25 ms) could be weakened. Actually, we could still observe AUC outliers at >0.25 ms jitter (Figure 3C). Here, we adopted a strict criterion to ensure validity; however, an appropriate jitter criterion that depends on the latency may enable more efficient identification.

#### Consistency with anatomical prediction

We compared identification rates per optical fiber among different patterns of interareal projections. The identification rate of commissural projections was significantly greater between symmetrical cortical areas than between asymmetrical areas (p = 1.7 × 10^−12^, *χ*^2^ test). In addition, the latency and jitter were significantly shorter within the same hemisphere than across hemispheres (p = 3.3 × 10^−9^ and p = 0.0081, respectively, rank-sum test). These results were consistent with the anatomical prediction from the amount, distance, and axonal properties of interareal projections.

#### Validation by cell types based on spike waveforms

**Figure 4:**
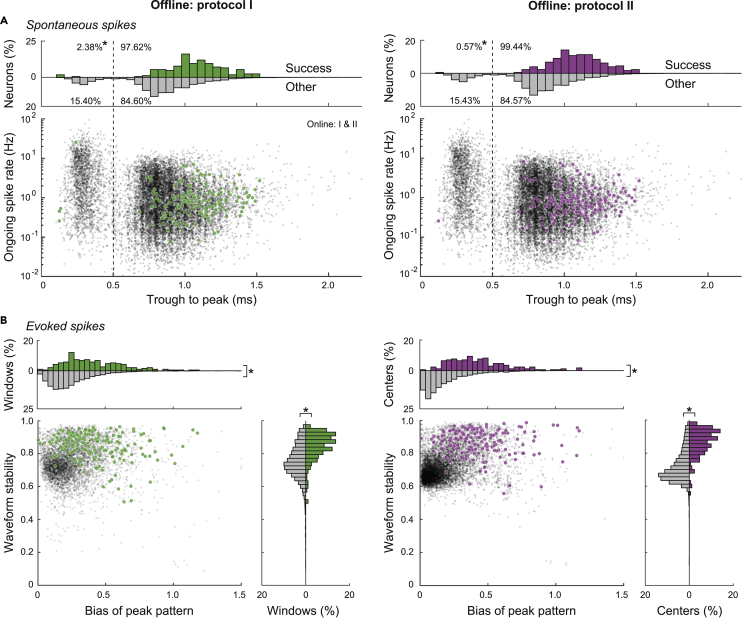
Comparison in spike properties between successes and others (A) Comparison in waveform durations of spontaneous spikes between successful (green and magenta dots) and other neurons (gray dots), in relation to subtypes of cortical neurons. We confirmed bimodal distributions (gray histograms on top) of the trough-to-peak durations of median spike waveforms and that the narrower type of cortical neurons exhibited higher ongoing spike rates than the wider type. Asterisks (∗) mean p < 0.005 in 2 × 2 *χ*^2^ tests for the fractions with a trough-to-peak longer than 0.5 ms, success vs. other. (B) Properties of inferred antidromic spikes to differentiate “success” from “other.” Each dot in the scatterplots represents the waveform stability and the peak pattern bias of an inferred group of antidromic spikes defined by the window (protocol I, left) and the center spike (protocol II, right) such that the jitter is sufficiently short (<0.25 ms). Asterisks (∗) mean p < 0.005 in rank-sum tests.

We also checked the cell types of successful neurons on the basis of the waveforms of spontaneous spikes. Fast-spiking neurons—a subtype of cortical neurons that exhibit narrow spike waveforms—are interneurons known to not project on other brain areas (Isomura et al., 2009). Actually, we observed a bimodal distribution of trough-to-peak durations in average spike waveforms of unsuccessful (other) neurons (gray dots and histograms in Figure 4A). The narrower group exhibited a higher ongoing spike rate, consistent with the properties of fast-spiking neurons. By contrast, successful neurons were scarcely found in the narrower group in judgment by either protocol I or II (green and magenta in Figure 4A, respectively). The fraction in the narrower group of successful neurons was smaller than that of other neurons (p = 3.3 × 10^−6^ in protocol I, p = 3.7 × 10^−8^ in protocol II, *χ*^2^ test for a spike duration <0.5 ms), suggesting that our judgment scarcely makes mistakes in adopting fast-spiking neurons as projection neurons.

#### Stability of evoked spikes

We next examined the properties of inferred antidromic spikes to differentiate successes from others. Even though the jitter of inferred antidromic spikes was sufficiently short (<0.25 ms), the inference might not always be true. Actually, there were numerous sets of inferred antidromic spikes for which no neuron was found to pass the collision tests. We attempted to find properties to improve the inference of antidromic spikes. If antidromic spikes could be stably evoked by stimulations at the target site, then the spike waveform patterns on four channels of the recording tetrode should be stable in every stimulation trial. We evaluated the waveform stability on the basis of trial-by-trial deviation from the median waveform pattern. The waveform stabilities of the successfully identified antidromic spikes were higher than those of the others (vertical values in Figure 4B; p = 4.1 × 10^−37^ in protocol I, p = 1.1 × 10^−60^ in protocol II, rank-sum test).

#### Multi-channel bias of evoked spike amplitudes

If synaptic spikes are evoked together in neuronal population around a tetrode, our inference of antidromic spikes by protocols I and II may adopt their population spike waves by mistake, which would exhibit an unbiased and stable peak pattern of four channels of the tetrode. By contrast, spike peak patterns of a single neuron would generally exhibit a bias depending on the direction of the neuron. Actually, we found that peak patterns of evoked spikes for successfully identified targets were more biased than those for the others (horizontal values in Figure 4B; p = 2.4 × 10^−37^ in protocol I, p = 1.1 × 10^−60^ in protocol II, rank-sum test). Most of the unsuccessfully identified targets were distributed around the region of low bias and stability, whereas the successfully identified targets ones scarcely fell around this region. This property implies that the online *Multi-Linc* controller might be improved by excluding hopeless targets of collision tests.

#### Similarity between trigger and evoked spike waveforms

**Figure 5:**
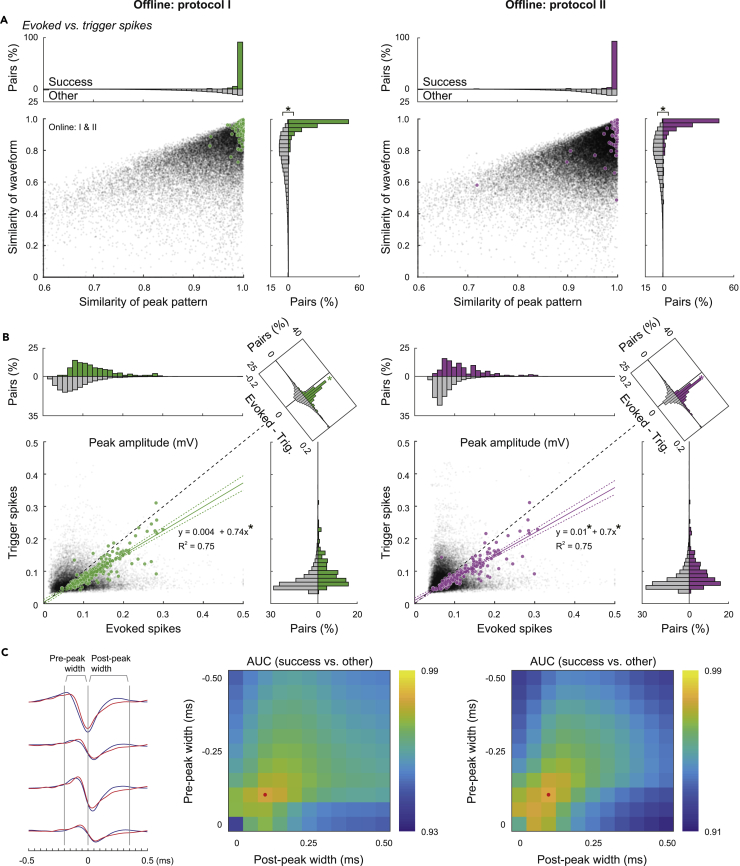
Similarity between trigger and evoked spike waveforms (A) Similarities in median waveforms between trigger and evoked spikes in the successful pairs in collision tests (green and magenta dots and histograms) and those in the other pairs (gray dots and histograms). Horizontal values represent the similarity for relative peak patterns on four tetrode channels (measured by direction cosines, common to the online controller), and vertical values represent the similarity for waveform patterns around peaks (−0.25 to +0.5 ms from the time point of the largest peak, 16 pt × 4 ch, measured by Pearson’s correlation coefficients; asterisks (∗) mean p < 0.005 in rank-sum tests for success vs. other). (B) Similarity between peak amplitudes of trigger and evoked spikes. The peak amplitude (largest of four channels) of trigger spikes in each successful pair was linearly correlated with that of evoked spikes (*solid lines*, linear regression; *dashed curves*, confidence intervals of 95%; asterisks (∗) mean p < 0.005 in linear regression tests and signed-rank tests for trigger vs. evoked). (C) Effective width of sampling points for waveform similarity to differentiate “success” from “other.” The combinations of pre-peak and post-peak widths (left) to calculate waveform similarity were swept in the range of 0-0.5 ms (peak ± 0-10 sampling points). The AUCs in ROC analyses for the waveform similarity of the successful pairs against the others are shown as a function of the pre-peak and post-peak widths. The maximum AUCs (red dots) were obtained at the widths of a few points (middle, peak ±2 pts in protocol I; right, peak ±1 pt in protocol II).

We next examined the relationships between trigger and evoked spike waveforms (Figure 5). We examined the similarity of the relative waveform patterns including several sampling points around the peak offline (vertical values in Figure 5A). The relative waveform similarity between trigger and evoked spikes of the successful pairs were greater than that of the other pairs (p = 9.7 × 10^−109^ in protocol I, p = 6.3 × 10^−99^ in protocol II, rank-sum test). We confirmed the similarity of trigger and evoked spikes in the successful pairs on the basis of the details of the waveform patterns.

Horizontal values represent the similarity of the relative peak patterns, which is the common index used to trigger collision tests in the online controller. The majority of successful pairs exhibited high peak-pattern similarity, confirming the condition used in the online controller (>0.99; 92% in protocol I, 94% in protocol II). This result suggests that the use of relative peak patterns worked well to trigger successful collision tests. However, successful pairs with lower peak-pattern similarity still existed. These pairs were picked by the offline analysis; they might not be targeted by the online controller. They also exhibited large similarities in waveform patterns; hence, the use of waveform patterns might improve the efficiency of the online controller.

Note that a substantial number of target pairs failed to pass collision tests even though the waveforms of trigger spikes and evoked spikes were similar (gray dots around [1,1] in Figure 5A). These target pairs would be regarded as light-responsive neurons in the widely used “phototagging” technique. Although unsuccessful pairs might contain insufficient trigger trials, we could find examples of sufficient trigger spikes that failed collision (Figure S6A), providing a warning that a short jitter does not always correspond to direct the antidromic activation of ChR2-positive neurons.

We next confirmed the relationships of the absolute amplitudes of trigger and evoked spikes. The amplitude of the evoked spikes on the peak channel was linearly correlated with that of trigger spikes in successful pairs (Figure 5B; p = 0.31, p = 4.7 × 10^−61^ for the zeroth and first orders in protocol I, p = 0.01, p = 6.3 × 10^−61^ for the zeroth and first orders in protocol II, *R*^2^ = 0.75 in both protocols, linear regression test), whereas the evoked spike sizes were larger than the trigger spike sizes (difference histograms in insets of Figure 5B; p = 1.5 × 10^−30^ in protocol I, p = 1.2 × 10^−30^ in protocol II, signed-rank test). This result might be attributable to the fact that spontaneous spikes are just initiated in the depolarized soma with high synaptic conductance, whereas antidromic ones reach full size after axonal propagation into hyperpolarized soma. Irrespective of the cause, we can utilize this property to improve the online *Multi-Linc* controller. The amplitude information can be used to trigger the collision tests by shrinking spike waveforms of evoked spikes with a certain ratio.

#### Efficient time points of waveforms to trigger collision tests

The online *Multi-Linc* controller utilized only the spike peak patterns to trigger the collision tests. If spike waveforms around the peaks are also utilized, then the performance might be improved. The waveforms up to 0.5 ms after the peak can be incorporated into real-time processing. We evaluated the segregation between successful pairs and others in the correlation coefficient of spike waveforms incorporating various patterns of pre-peak and post-peak widths (Figure 5C). The segregation was quantified by AUC of ROC analyses of the correlation coefficients. In judgment by either protocol I or II, the optimal segregation was obtained in waveform patterns within peak ±0.15 ms and the waveform of peak ±0.05 ms (three points of peak ±1 at 20 kHz sampling) exhibited sufficient segregation (AUC = 0.98 in protocol I, AUC = 0.98 in protocol II). The use of waveform similarity of a few time points around the peak can improve the efficacy in triggering the collision tests.

### Simultaneous identification of reciprocal projections

**Figure 6:**
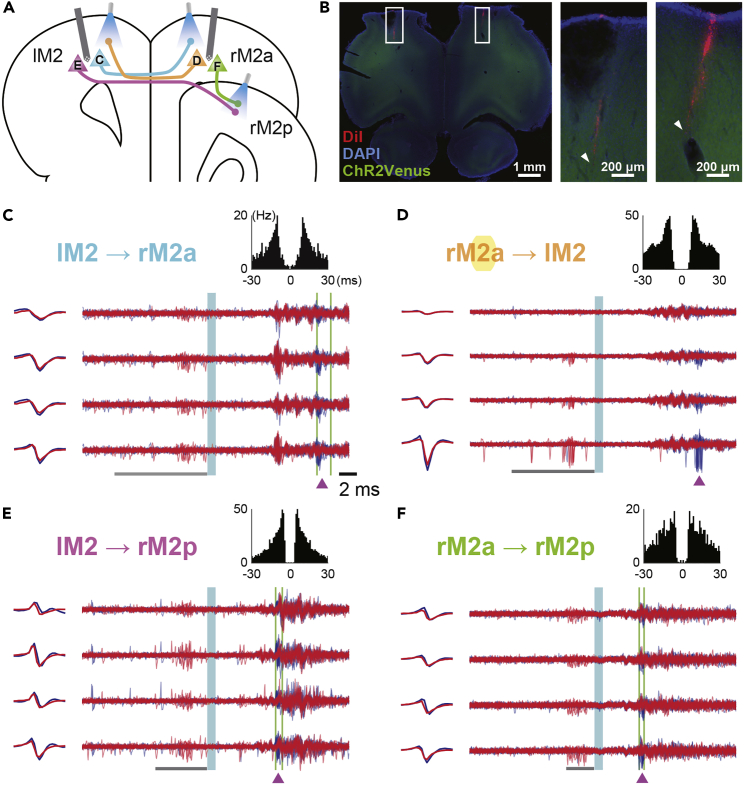
Simultaneous spike collision tests in reciprocally connected areas (A) Coronal view of bilateral recording and stimulation sites in the secondary motor cortex (M2). Neurons C-F correspond to panels (C-F) below, respectively. (B) Histological confirmation of silicon probe insertion. The right two photos (magnified from the white boxes at left) indicate insertion tracks (arrowheads) marked with red fluorescent dye DiI (blue, DAPI; green, ChR2-Venus). (C-F) Successful spike collision tests by the *Multi-Linc* system, which simultaneously identified four neurons projecting from left M2 (lM2) to right M2, anterior (rM2a) (C), from rM2a to lM2 (D), from lM2 to right M2, posterior (rM2p) (E), and from rM2a to rM2p (F). The corresponding data are shown in Figure 3A.

One of the advantages of our *Multi-Linc* method is the ability to simultaneously identify multiple patterns of projections among different areas in every recording session. Even if these projections are reciprocal and overlapped with observed neurons, they can be identified independently with no interference. Such reciprocal connections are known to play pivotal roles in the coordination of brain functions: e.g., commissural interaction between hemispheres and feedforward-feedback interaction in hierarchical areas. Other standard methods, such as calcium imaging and “optotagging,” utilize projection-specific expression of genetically encoded calcium indicators (GECIs) or optogenetic opsins through viral vector infection. For reciprocal connections, however, the specificity of fluorescence measurement or photoactivation to each projection may not be guaranteed even using two distinct color indicators/opsins because one-expressing neurons are overlapped with another-expressing background tissue in the vector injection site (see discussion). To show this prospect of our *Multi-Linc* method, we attempted to apply both recording and stimulation for bilateral motor cortices. We successfully obtained examples of reciprocal projections and projections to different target areas (Figure 6). We also confirmed that the online *Multi-Linc* controller worked with virus injection to express ChR2 in neurons of a wild-type rat (Figures S6B−S6D).

## Discussion

### Summary

We implemented an online (real-time) controller system to automate and parallelize the spike collision tests (Figure 2). We attempted multiple protocols (protocols I and II) in the online controller of closed-loop experiments to infer the antidromic spikes because there has been no previous attempt to automate and parallelize the spike collision tests. We cross-checked both data in the offline judgements by protocols I and II. As the results of offline judgements, we found simultaneous identifications of multiple projections in many sessions by both protocols I and II (maximum of 13 projections in a session; Figure S3), which contained examples of reciprocal projection patterns (Figure 6). These were validated by the different protocols in the offline judgements from those in the online controller (cross-checking). The outcomes of the two protocols were comparable. Hence, both protocols are prospective for future development.

The main advantage of our *Multi-Linc* analysis is the potential to simultaneously identify spike-timing signals through interareal projections of many sources and targets, including reciprocal patterns, at arbitrary depths from the brain surface. Although large-scale identification in deep brain areas has a limitation owing to the physical or invasive constraint to penetrate electrical probes and optical fibers, the limitation will be weakened by the development of micro-and-concentrated probes and fibers.

In our *Multi-Linc* analysis, neuronal projections with larger spike amplitudes are apt to be identified. Although this kind of selection bias is common to all analyses depending on the extracellular electrophysiological recording, we seek reliable identification rather than eliminating the selection bias in antidromic spike inference. The bias will be dissolved by imaging techniques with a sufficient temporal resolution to detect spike signals.

### Comparison with other methodologies

Thus far, several methodologies have been used to examine functional spike signals between brain areas in behaving animals. Juxtacellular recording, a conventional electrophysiological technique (Pinault, 1996), enables spike measurement and *post hoc* visualization of a single neuron, which can be used to track its axonal projection histologically. However, juxtacellular neuron identification consumes large numbers of valuable task-trained animals because only a few neurons can be visualized histologically from each animal (Isomura et al., 2009, 2013).

Specific gene expression via retrograde viral vectors (Kato et al., 2011; Tervo et al., 2016) is useful in examining the functional activity of numerous projection neurons, especially when used in combination with two-photon laser-scanning or microendoscopic calcium imaging techniques (e.g., Osakada et al., 2011; Ren et al., 2018) with genetically encoded calcium indicators (GECIs) (Nakai et al., 2001; Zhao et al., 2011; Chen et al., 2013). Calcium imaging captures relative changes in the spike rate with low temporal resolution but not precise spike events. Hence, analyzing spike correlation with other neurons or with neural oscillations such as theta and gamma waves is almost impossible (Isomura et al., 2006; Sirota et al., 2008). Moreover, only a few projection pathways can be distinguished even by the latest multi-color calcium imaging techniques with different GECIs (Inoue et al., 2019) from retrograde viral vectors. In addition, the expression of GECIs takes at least several weeks after viral injection to the target areas.

In recent years, the “optotagging” (phototagging) technique has been widely used to identify neuron subtype or axonal projection by specifically stimulating ChR2-expressing neurons optogenetically (Jennings and Stuber, 2014; da Silva et al., 2018; Starkweather et al., 2018). Typically, retrograde/anterograde viral vectors enable projection-specific expression of optogenetic opsins. Identification of multiple projections requires local vector injections of two or three different opsins with their effective wavelengths separated sufficiently. It is still difficult to stimulate one specifically in reciprocal projections at the vector injection site. Moreover, in the case where excitatory glutamatergic neurons are designed to express ChR2 for optotagging, unrelated ChR2-negative neurons could also respond to light stimuli synaptically through excited neurons (see Figure 1). Such false-positive neurons cannot be excluded by optotagging alone even if spike jitter is sufficiently small (see Figures 3C and S6A). Thus, projection-specific expression does not always mean projection-specific excitation, which is why the spike collision test is still necessary for electrophysiological identification of axonal projection.

### Improvement from conventional spike collision tests

The greatest drawback of the spike collision test is its inefficiency. Here, we have paved the way to overcome this drawback by automating and parallelizing multi-channel spike collision tests. Our new *Multi-Linc* analysis has practical advantages over other methods in the exploration of projection neurons. Using transgenic animals expressing ChR2 broadly in the brain (Tomita et al., 2009; Saiki et al., 2018), we can flexibly test spike collisions in a different combination of multiple stimulating and recording sites in each experiment. The method is not restricted by the number of excitation wavelengths or by the sites and timing of viral injection. The method should also be easily applicable to long-distance pathways in the primate brain, which ensures sufficient spatiotemporal separation between stimulation and recording. Because of the versatility of the *Multi-Linc* analysis method, we expect to simultaneously observe cooperative or feedforward-feedback signaling through reciprocal projections between areas (see Figure 6).

### Potential improvements in the future

Nevertheless, our *Multi-Linc* analysis still has substantial room for improvement in its efficiency and accuracy. First, because ChR2 is expressed throughout the neuron, antidromic spike signals would be contaminated with noisy spikes by stimulating somata and dendrites of other neurons or a false projection could be identified because of incorrect stimulation of the midway of its axon. To avoid these problems, we developed a ChR2 variant that preferentially localizes at axonal terminals and that is available for spike collision tests (Hamada et al., 2021). Such ChR2 optimization for *Multi-Linc* analysis will lead to improvements in its temporal efficiency and spatial accuracy. Second, the inducibility of antidromic spikes depends on the properties of axons and the degree of ChR2 expression, which could result in selection bias of neurons for testing (Swadlow, 1998). Such bias will be eliminated by sufficient ChR2 expression in every axon and by local laser stimulation. Third, additional neurons would be assayed accurately by developing a real-time closed-loop system with high-density multi-channel probes (e.g., Neuropixels; Jun et al., 2017; Steinmetz et al., 2019). If spikes can be optically imaged *in vivo* using genetically encoded voltage indicators (Gong et al., 2015), an “electrodeless” *Multi-Linc* analysis could be developed in the future. Third, we might miss projecting neurons with short latency because of the neuronal refractory period and the evoking delay of ChR2 (see STAR Methods). The limitation caused by the neuronal refractory period originates in the principle of collision tests (Swadlow, 1998). However, if we optimize the exclusion criterion of trials to avoid false positives by estimating the refractory period for each neuron, identification efficacy for short-latency neurons will be improved. Actually, we confirmed that the relaxation of this criterion increases the number of identified projections (Figure S3C). Missing projecting neurons owing to the evoking delay can also be reduced if an opsin to evoke spikes more quickly is developed. Lastly, the algorithm for *Multi-Linc* analysis needs to be further improved to enhance its performance (e.g., to achieve precise detection of evoked spikes on the basis of the all-or-none law, optimal prioritization of neurons to be tested, and a stimulation protocol that does not interfere with brain states and behavior). These improvements could, eventually, lead to a “high-throughput” *Multi-Linc* analysis to elucidate the principle of spike communication among brain areas.

### Limitation of the study

• The minimum inter-stimulation interval to avoid epileptic neuronal responses (∼0.5 s) is a bottleneck to gathering trials of spike collision tests per unit time. This limit might be overcome by the optimization of the stimulation patterns.
• Real-time acquisition of spike recording to a general-purpose computer is limited. The limit of our system is a maximum of 128 recording channels. This limit might be overcome by implementing our algorithms into specific-purpose hardware (e.g., a field-programmable gate array (FPGA)).
• The number of multiple probes and optical fibers has a physical limit for insertion into the brain. The limitation will be weakened by the development of micro-and-concentrated probes and fibers.

## STAR★Methods

### Key resources table

**Table:** 

REAGENT or RESOURCE	SOURCE	IDENTIFIER
**Antibodies**		

Anti-GFP, polyclonal, rabbit/IgG	invitrogen	Catalog #: A6455, RRID: AB_221570
Alexa Fluor^TM^ 488, Anti-rabbit, polyclonal, donkey/IgG	invitrogen	Catalog #: A21206, RRID: AB_2535792

**Bacterial and virus strains**		

AAVDJ-Syn-hChR2-EYFP, 1.8 × 1010 vg/μL, 1 μL/site	Gift from Dr. Kenta Kobayashi at the National Institute for Physiological Sciences, Japan	N/A

**Deposited data**		

Repository Data	This paper	https://github.com/sakai-tamagawa/MultiLinc

**Experimental models: Organisms/strains**		

Rat: Iar:Long-Evans, wild-type	Japan SLC	http://www.jslc.co.jp/animals/rat.php
Rat: Iar:Long-evans, Thy1.2-ChR2V4 tg	Tomita et al., 2009	N/A

**Software and algorithms**		

EToS	Takekawa et al., 2012	http://etos.sourceforge.net
Matlab2019a	Mathworks	RRID:SCR_001622
Original code	This paper	https://github.com/sakai-tamagawa/MultiLinc

**Other**		

Interface (ACQ2106)	D-TACQ Solutions	
Laser system (MiLSS, custom -made)	ASKA	
Silicone probes (ISO_3x_tet_A32)	NeuroNexus	
Optical fiber (FT1000EMT, diameter: 1000 μm)	Thorlabs	https://www.thorlabs.co.jp/thorproduct.cfm?partnumber=FT1000EMT
Real-time computer (Intel Xeon Silver 4110 CPU @ 2.10GHz, 8 cores; Linux OS, Ubuntu 14.04, real-time kernel)	D-TACQ Solutions	

### Resource availability

#### Lead contact

Further information and requests should be directed to and will be fulfilled by the lead contact, Yutaka Sakai (sakai@tamagawa.ac.jp).

#### Material availability

This study did not generate new unique reagents.

### Experimental model and subject details

#### Animal preparation

All experiments were approved by the Animal Research Ethics Committee of Tamagawa University (Animal Experiment Protocol H30-32) and the Institutional Animal Care and Use Committee of Tokyo Medical and Dental University (A2019-274) and were carried out in accordance with the Fundamental Guidelines for Proper Conduct of Animal Experiment and Related Activities in Academic Research Institutions (Ministry of Education, Culture, Sports, Science and Technology of Japan). All surgeries were performed under appropriate isoflurane anesthesia, and all efforts were made to minimize animal suffering. The procedure for animal experiments was established in a series of studies by our group (Isomura et al., 2009, 2013; Soma et al., 2017; Nonomura et al., 2018; Saiki et al., 2018; Rios et al., 2019; Kawabata et al., 2020). We used eight adult male rats (407.6 ± 32.2 g; six Wister Thy1.2-ChR2V4 line backcrossed with the Long–Evans strain (Tomita et al., 2009; Saiki et al., 2018); two wild-type Long–Evans strain (Sankyo Labo Service, Tokyo, Japan) for viral vector injection). These rats were kept under an inverted light schedule (lights off at 12 AM; lights on at 12 PM) in their home cages to adapt to experimental surroundings.

#### Surgery

Primary surgery was performed to attach a head-plate (CFR-2, Narishige, Tokyo, Japan) to the skull of rats under anesthesia by isoflurane gas (4.0–4.5% for induction and 2.0–2.5% for maintenance, Pfizer Japan, Tokyo, Japan) using an inhalation anesthesia apparatus (Univentor 400 anesthesia unit, Univentor, Zejtun, Malta). The body temperature was maintained at 37.0°C using an animal warmer (BWT-100, Bio Research Center, Aichi, Japan) during anesthesia. The head of rats was fixed on a stereotaxic frame (SR-10R-HT, Narishige) with ear bars, and applied with lidocaine (Xylocaine Jelly, Aspen Japan, Tokyo, Japan) for local skin anesthesia and povidone-iodine disinfectant solution (10%, Kaneichi, Osaka, Japan) for disinfection around surgical incisions. The head-plate was then glued to the skull with stainless steel screws and dental resin cement (Super-Bond C & B, Sun Medical, Shiga, Japan; Unifast II, GC Corp., Tokyo, Japan), and reference and ground electrodes (PFA-coated silver wires, A-M systems, WA; 125-mm diameter) were implemented under the bone on the cerebellum. Analgesics and antibiotics (meloxicam, 1 mg/kg sc, Boehringer Ingelheim, Tokyo, Japan; gentamicin ointment, 0.1% us. ext., MSD, Tokyo, Japan) were finally applied to remove pain and prevent infection.

More than 1 week later, secondary surgery was performed to make cranial windows to frontal motor cortices bilaterally (1.0–3.5 mm anterior and ±1.0–3.0 mm lateral from the bregma) under the isoflurane anesthesia. The bone and dura mater were opened and removed by a dental drill (Tas-35LX, Shofu, Kyoto, Japan) and a dura picker (DP-T560-80, Bio Research Center, Aichi, Japan). The cortical surfaces were washed with PBS containing antibiotic (0.2% amikacin sulfate, Sawai, Osaka, Japan) and covered with antibiotic ointment (Chlomy-P ointment AS, Daiichi Sankyo Healthcare, Tokyo, Japan) and dental silicone sealant (DentSilicone-V, Shofu, Kyoto, Japan) until recording experiments.

### Method details

#### System for online *Multi-Linc* procedure

##### Hardware

We performed *Multi-Linc* experiments with a closed-loop controller for multi-channel recording and optogenetic stimulation. The online controller system was composed of a real-time processing computer [Intel Xeon Silver 4110 CPU @ 2.10GHz, 8 cores; Linux OS, Ubuntu 14.04, real-time kernel; D-TACQ Solutions, Scotland, UK] connected to a set box for input and output interfaces (D-TACQ ACQ2106; 128 channels of 16-bit analog inputs and 32 channels of digital-TTL outputs). In the system, data acquired through the 128 input channels at 20 kHz synchronous sampling were assured to transfer every 1.6 ms to the shared memory available on the CPU of the computer and arbitral TTL patterns of 20 kHz clocks set on the shared memory were assured to transfer every 1.6 ms to the output terminals. The analog input channels are supposed to receive amplified multi-channel signals from silicon probes inserted into the brain. The digital output channels are supposed to send signals to a multi-fiber laser stimulation device for optogenetic stimulation.

For analog inputs from multi-channel recording, we used 32-channel silicon probes (ISO_3x_tet_A32; with seven tetrode-like electrodes on three shanks, NeuroNexus, Ann Arbor, MI). The signals from the silicon probes were amplified 2000 times and bandpass-filtered between 0.5 Hz and 10 kHz through pre- and main analogue amplifiers (MPA32I and FA32I, Multi Channel Systems, Reutlingen, Germany).

For optogenetic stimulation through digital TTL outputs, we used a multi-fiber laser stimulation device (MiLSS, custom-made; ASKA, Hyogo, Japan) to emit a blue light pulse (445 nm, 7–15 mW at the end) into each of seven optical fiber ports with a two-axis galvanometer mirror under the control of 8-channel TTL signals. The light pulse was delivered after the laser stimulation device started emitting the laser beam (1 ms delay) and the galvanometer mirror moved (as long as 0.75 ms).

The multi-channel inputs and TTL outputs were bifurcated to sets of 32-channel digital recording devices (LX-120, TEAC, Tokyo, Japan; 16 bit, 20 kHz) for the offline *Multi-Linc* procedure after the experiments.

##### Software

We composed the controller software of a web-based user interface (UI) by CGI (Common Gateway Interface; Perl 5, lighttpd 1.4) and process commands implemented in the C and C++ languages controlled by shell scripts. Experimenters can manipulate the system through standard web browsers on the local network by setting parameters and sending start and stop signals for the respective procedures. The commands for start and stop signals are called by CGI form. Once the main process starts, multiple commands run and interact in real time through the shared memory.

##### Real-time filtering and spike detection

We implemented simple high-pass filtering for real-time spike signal detection with a one-side exponential filter:

$$z_{c}\left(t\right)=x_{c}\left(t\right)-y_{c}\left(t\right)\text{,}$$

$$y_{c}\left(t+1\right)=y_{c}\left(t\right)+G_{y}z_{c}\left(t\right)\text{,}$$

where $x_{c}\left(t\right)$ is the local field potential acquired from channel *c* at time point *t*, $y_{c}\left(t\right)$ is the local average, and $z_{c}\left(t\right)$ is the filtered value. The parameter $G_{y}$ determines the averaging scale (default 0.25 ms corresponds to $G_{y}$= 0.2 at 20 kHz sampling). Spike detection was based on the noise level on a tetrode of four channels. To avoid square-root computations at every step, we compared the square summation of four channels with the noise variance. Because a spike signal appears to be a negative sharp peak in extracellular recoding, we considered only the negative components,

$${\hat{z}}_{c}\left(t\right)=\min\left(z_{c}\left(t\right),0\right)\text{,}$$

$$a\left(t\right)=\sum_{c=1}^{4}{\hat{z}}_{c}^{2}\left(t\right)\text{,}$$

$$v\left(t+1\right)=v\left(t\right)+G_{v}\left(a\left(t\right)-v\left(t\right)\right)\text{,}$$

$$a\left(t\right)>\theta_{\text{sp}}^{2}v\left(t\right)\;\Rightarrow spike\;on\text{,}$$

where a(t) is the square amplitude of the rectified four-channel vector of a tetrode and v(t) is an estimate of the amplitude variance. The parameter $G_{v}$ determines the averaging scale of the variance estimation (default 1 sec corresponds to $G_{v}=5\times 10^{-5}$). The parameter $\theta_{\text{sp}}$ is the threshold of spike detection relative to the noise level ($\theta_{\text{sp}}=$ 4 [SD] at default). The peak time point in the range of successive “spike on” is defined as the spike time $t_{k}$, and the four-channel vector at the time, ${\hat{z}}_{k}=\left({\hat{z}}_{1}\left(t_{k}\right),\;\ldots ,\;{\hat{z}}_{4}\left(t_{k}\right)\right)$ and the squared amplitude $a_{k}=a\left(t_{k}\right)$, are stored in the shared memory. The real-time filtering and spike detection always run for every tetrode parallelly from the session start to the end.

##### Evoked response collection

A stimulation site is serially selected and the stimulation signal is sent through the shared memory at a random timing such that inter-stimulation intervals might be longer than the criterion (i.e., the minimum interval between the same-site stimulations, *I*_same_) and between the different-site stimulations, *I*_diff_ (*I*_same_ = 1.0 sec, *I*_diff_ = 0.5 sec at default). These minimum intervals are set to avoid epileptic neural responses. The duration of a stimulation (TTL on) is set via parameter *D*_*i*_ for each stimulation site *i* (default: 1.0 ms). Experimenters can manipulate the duration of each site through pre-session tests. After every stimulation, raw data $\left\{x_{c}\left(t\right)\right\}$ from all tetrodes during a time range *T*_range_ (default: 30 ms) before and after the stimulation are stored from the ring buffer on the shared memory. While waiting the minimum inter-stimulation intervals, the stored $\left\{x_{c}\left(t\right)\right\}$ data are filtered semi-offline with a precise symmetric filter common to the offline analyses described in the section of the offline *Multi-Linc* procedure. The high-pass filter is designed to subtract Gaussian-smoothed signals (*σ* = 0.25 ms). The precisely filtered data are stored in the files for use in the next process. The process of the evoked response collection stops so the next process can be started when the required number of stimulations per stimulation site, *N*_anti_ (default: 100) have been collected.

##### Antidromic spike inference

After the evoked responses are collected, the process of antidromic spike inference starts semi-offline. The precisely filtered evoked responses of each tetrode are normalized into the Z-score based on the estimated noise level during the range *T*_range_ before stimulations. The noise level is estimated for each tetrode commonly among four tetrode channels by averaging over the stimulation trials of all the stimulation sites. Antidromic spikes in evoked responses are inferred for each tetrode–stimulation site pair. We used two different inference protocols (protocols I and II, see Figure 2). The inference protocols were common to the offline analyses. The details of the inference protocols are described in the section of the offline *Multi-Linc* procedure. The averaged peak amplitude patterns of inferred antidromic spikes are transferred to the next process.

During the semi-offline process for the antidromic spike inference, frequency-following tests can be optionally executed as a parallel process. Several repetitive stimulations with short intervals are executed for every stimulation site. Because ChR2 itself cannot respond at such a short interval (Gunaydin et al., 2010), the frequency-following test is not effective to verify the collision test in the present experimental paradigm. Therefore, we implemented it as an option and confirmed that it could work (Figure S6E).

##### Collision test

The process of the collision test monitors spike occurrence on all tetrodes in real time through the shared memory. When a spike is found on a certain tetrode, the targets associated with the tetrode and the stimulation sites such that the intervals from the last stimulations are longer than the criteria of the minimum inter-stimulation intervals, *I*_same_ and *I*_diff_, are sought. The squared direction cosine to each targeted peak pattern is calculated as the similarity to the inferred antidromic spikes, $D={\left({\hat{z}}_{k}\cdot u_{j}/\left|u_{j}\right|\right)}^{2}/a_{k}$, where $u_{j}$ is the peak pattern vector of the *j*-th target for collision tests. If the similarity is greater than the criterion, $D>\theta_{\text{trig}}^{2}$ (default: $\theta_{\text{trig}}=0.99)$, then the spike triggers a collision test for the *j*-th target and the stimulation signal of the corresponding site is immediately set in the shared memory. Packet communication every 1.6 ms with the input–output interfaces assures a TTL onset within 3.2 ms after the spike. If the number of collision tests for a target reaches the criterion, *N*_test_ (default: 200), then the target is excluded. The process stops if all targets are excluded, or if the experimenter sends the stop signal.

#### Experimental data collection

##### Multi-channel recording and optogenetic stimulation

We performed online *Multi-Linc* experiments in the frontal motor cortices of unanesthetized rats under head-fixation. For the multi-channel recording, we used two 32-channel silicon probes, although the system can accommodate 128 channels. Approximately 1 h before each recording session, the probes were inserted to a depth of 1.0–1.5 mm from the cortical surface, typically in layer 5, where intratelencephalic (IT)-type projection neurons are distributed most abundantly, using three-axis micromanipulators (SMM-200B and SMM-100, Narishige). On the last recording day, the probe tracks were marked with the red fluorescent dye DiI (DiIC18(3), PromoKine, Heidelberg, Germany) applied to the back of each shank for histological conformation. In the optogenetic stimulation, we used micromanipulators (SM25A, Narishige) to place two to six optic fibers (FT1000EMT, diameter: 1000 μm, Thorlabs, New Jersey, USA) on the cortical surface in a symmetrical position contralaterally from the silicon probes.

##### Histology

After all recording sessions, the rats were perfused transcardially with cold saline and subsequent 4% formaldehyde in 0.1 M phosphate buffer under deep anesthesia with urethane (3 g/kg, ip, Nacalai Tesque, Kyoto, Japan) to confirm the final probe tracks. The brains were removed and post-fixed at least overnight, and 50 μm-thick serial coronal sections were prepared using a microslicer (VT1000S, Leica, Wetzlar, Germany). The serial sections were cover-slipped with mounting medium (DAPI Fluoromount-G, Southern Biotech, AL, USA) and observed under a fluorescence microscope (IX83 inverted microscope, Olympus, Tokyo, Japan).

#### Offline *Multi-Linc* procedure

##### High-pass filtering and spike sorting

Signals from each tetrode channel were filtered with a high-pass filter designed to subtract Gaussian smoothed signals (*σ* = 0.25 ms) throughout an experimental session (several hours). The filtered data were normalized into Z-scores on the basis of the common noise level estimated for each tetrode among four tetrode channels throughout the session. Spike events of individual neurons were isolated and clustered in each tetrode using the automatic spike sorting software EToS (Takekawa et al., 2012) and the manual spike clustering software Klusters (Hazan et al., 2006; Figure S2D).

##### Antidromic spike inference in protocol I

Filtered responses on a certain tetrode evoked by stimulations to a certain site were aligned with the onset of stimulation. The temporal window of a fixed width (2 ms width in the online experiments, tuned to 1 ms in the offline analyses) was slid along time from channel 1 to 4 (Figure 2B, top), and the negative peak within the window was detected in each trial (Figure S2A). For each window, the distribution of the negative peaks was obtained (dots in Figure 2B, middle). The goodness of the window can be characterized by the first quartile (75% from the largest) of the amplitude distribution because a larger amplitude quartile indicates a more stable occurrence of spikes within the window (Figure S2B). All the windows of timings and channels were characterized in the same manner. The windows with the amplitude quartiles below a certain threshold were excluded from candidates. Among the remaining candidates, the window with the maximum amplitude quartile was adopted as the best window (Figure 2B, bottom, and Figure S2C). Once adopted, the window width was fitted to the jitter distribution. Using 75% from the largest peak, the jitter was estimated as the quartile deviation QD of the peak timings. The window start and end were reset by multiplying a factor *W*_jitter_ to the deviation from the median timing as median ± *W*_jitter_ QD (*W*_jitter_ = 5, online; *W*_jitter_ = 4, offline). The distribution of negative peaks within the new window was determined again, and the window start and end were reset in the same manner. If the window converged after the iteration, we adopted the window to determine inferred antidromic spikes. We determined 75% from the largest peak within the converged window as representative spikes inferred to be antidromic. The median waveform of each tetrode channel was calculated among the representative spikes at each time point relative to the peak timings. After an inferred window was adopted, the sliding windows on all channels overlapping the time range of the adopted window were excluded from candidates, and the selection of an inferred window among the remaining candidates was iterated.

##### Antidromic spike inference in protocol II

The protocol II evaluated the aggregation of timings and waveform features of spikes. First, spikes in evoked responses were detected by thresholding (negative peaks below 5 SD). We defined the similarity between two evoked spikes as

$$S_{kl}=\text{exp}\left[{\left(\frac{{\hat{z}}_{k}・{\hat{z}}_{l}}{\left|{\hat{z}}_{k}\right|\left|{\hat{z}}_{l}\right|}\right)}^{2}-1-\;\frac{{\sin\;}^{2}\left(\pi /18\right)}{\alpha^{2}}{\left(t_{k}-\;t_{l}\right)}^{2}\right]\text{,}$$

where ${\hat{z}}_{k}$ and *t*_*k*_ are the four-dimensional vector of the *k*-th spike amplitude pattern on the tetrode channels and the timing from the stimulation onset, respectively. Spike amplitudes were rectified to be non-positive values, and the angle difference of two vectors was smaller than *π*/2; hence, the similarity decreased monotonically with increasing angle difference. The similarity of identical spikes gives the maximum value *S*_*kk*_ = 1. The parameter *α* controls the weight of the timing deviation. The timing deviation *t*_*k*_ − *t*_*l*_ = *α* corresponds to the angle difference of *π*/18 between peak amplitude directions. The acceptable jitter *α* was tried in the range 0.1–4 ms for the online controller and 1 ms for the offline analyses.

We searched the center of aggregated spikes among all trials of stimulations on the basis of the aforementioned similarity. We focused on each detected spike as a candidate for the aggregation center and determined the spikes most similar to the focused spike in respective trials (Figure S2A). The degree of aggregation around the focused spike can be characterized by the first quartile in the similarity distribution (75% from the most similar one; Figures 2C and S2B). All detected spikes were characterized by the similarity quartiles in the same manner. Spikes with similarity quartiles less than a threshold θ_aggr_ were excluded from candidates for the center spike (online default: θ_aggr_ = 0.98; candidates were not restricted in the offline analyses to gather control samples). Among the remaining candidates, the center spike with the maximum similarity quartile was adopted as the aggregation center (Figure 2C, bottom, and Figure S2C). As in protocol I, we used 75% of the nearest spikes to the adopted center spike as representative spikes inferred to be antidromic. The median spike waveform and the median timing were calculated among the representative spikes. Once adopted, 75% of the nearest spikes were excluded from candidates for the next center spike. Selection of the center spike was then iterated.

##### Judgement in collision tests

In offline analyses, we tested all the pairs of neurons (spike clusters) and inferred sets of antidromic spikes (defined by windows in protocol I, and center spikes in protocol II) for whether spike collisions might occur. Two criteria were used for the determination (Figures 3C and S1A). The first criterion was based on the receiver operating characteristic (ROC) analyses to evaluate the elimination of evoked spikes by collisions. The second criterion was the jitter of inferred antidromic spikes.

If spontaneous spikes occur immediately before the timing of spike to be evoked, the evoked spike can be eliminated by the neuronal refractory period rather than the collision (see Figure 1B). The range of spike elimination by the refractory period can be described as *t*_stim_ + *L* = *t*_stim_ + *d* + *C* < *t*_spon_ + *R*, where *L* is the latency of an evoked spikes at the recording site from the stimulation timing, which consists of the evoking delay *d* at the stimulation site and the conduction time *C* through the axon (Bishop et al., 1962; Fuller and Schlag, 1976; Lipski, 1981). The timing of the stimulation and the spontaneous spike are denoted by *t*_stim_ and *t*_spon_, respectively, and *R* is the refractory period of the neuron. Spike elimination by the refractory period can occur if the evoked spike is synaptic rather than antidromic. To avoid false-positive identification strictly, we excluded such trials with the strict criterion, that is, we excluded trials such that spontaneous spikes occurred within the possible supremum of refractory periods before the earliest timing of the inferred antidromic spikes. This range can be described as *t*_min_ − *R*_max_ < *t*_spon_, where *t*_min_ is the earliest timing of the inferred antidromic spikes (see Figure S1B), and *R*_max_ is the possible supremum of refractory periods supposed in neurons of the recording site. We adopted *R*_max_ = 4 ms on the basis of juxtacellular recording in the same recording area (*R* = 2.8 ± 1.2 ms in Isomura et al., 2009).

For each pair of a neuron and an inferred set of antidromic spikes, the trigger and no-trigger stimulation trials were extracted from the remainder of the stimulation trials. The trigger trials were defined such that spikes of the neuron occurred in the trigger range before the stimulations. The trigger range was defined such that a spontaneous spike within the range should collide with the antidromically evoked spike (see Figure S1B). Spike collision does not occur if the spike evoked timing misses the arrival of a spontaneous spike at a stimulation site, *t*_spon_ + *C* + *R’* < *t*_stim_ + *d*, where *R*′ is the refractory period of an arriving spike on the axon (Swadlow, 1982). We could not record the evoking delay *d*, but as long as the antidromic spikes are evoked stably, the spikes would be evoked before the offset of stimulation light, because ChR2 would be closing after the offset. Using inequalities *t*_stim_ + *d* < *t*_off_, *t*_min_ − *t*_off_ < *C*, and 0 < *R′*, the condition of possible missing is obtained as *t*_spon_ < *t*_off_ + *t*_off_ − *t*_min_ = *t*_off_ − *L*_min_, where *L*_min_ = *t*_min_ − *t*_off_ and *t*_off_ is the timing of stimulus offset. Thus, we defined the trigger range [*t*_off_ − *L*_min_, *t*_stim_] to exclude possibly missing trials. If the number of the extracted trigger trials was less than 15, then the focused pair was excluded from the targets of collision tests.

If the trigger trials were defined, then we extracted no-trigger trials in which no spikes of the neuron occurred in the no-trigger range before the stimulations from the trials near the trigger trials. The no-trigger range was defined such that a spontaneous spike within the range could collide with the antidromically evoked spike (see Figure S1B). Contrary to the trigger trials, possible collision should be excluded. Using inequalities 0 < *d*, *C* < *t*_max_ − *t*_stim_, and *R’* < *R*_max_, the condition of the possible collision is obtained as *t*_spon_ > *t*_stim_ + *t*_stim_ − *t*_max_ − *R*_max_ = *t*_max_ − *L*_max_ − *R*_max_, where *L*_max_ = *t*_max_ − *t*_stim_ and *t*_max_ is the latest timing of the inferred antidromic spikes. Thus, we defined the no-trigger range [*t*_stim_ − *L*_max_ − *R*_max_, *t*_stim_] to exclude possible collision. We extracted the nearest 10 no-trigger trials per each trigger trial, and merged trials duplicated among different trigger trials. Thus, the number of extracted no-trigger trials was ten times the number of trigger trials at maximum.

We evaluated the elimination of spikes to be evoked with negative peak amplitudes within the inferred window in protocol I and the similarities to the inferred center spike in protocol II (Figures S1C, S1D, 2E and 2F). The ROC analysis was performed between distributions of the evaluated variables (peak amplitudes or similarities) in the extracted trigger and no-trigger trials, and the area under the ROC curve (AUC) was calculated. The criterion for spike elimination was defined on the basis of the AUC distribution among the whole tested pairs such that AUC might be more than five times the standard deviation σ robustly estimated with the relation in the Gaussian distribution, *σ* = median[|AUC−median[AUC]|]/0.6745. The jitter was defined as the quartile deviation of the latencies of evoked spikes determined by the inferred window (protocol I) or center spike (protocol II) in no-trigger trials. Notably, the latency of each spike was calculated by spline interpolation for its waveform.

If AUC > 5*σ* and jitter <0.25 ms, then the tested pair was defined to be successful in the collision test. If multiple successful pairs were found for an identical neuron, then only the pair with the highest AUC was adopted as a successful pair.

#### Analysis for the spike property of identified projection neurons

##### Spontaneous spikes

We computed the trough-to-peak durations of unfiltered and interpolated waveforms and the ongoing spike rate of spontaneous spikes for each neuron in the range from the first to the last stimulation in a session, except for a 30 ms period following each stimulation (Figure 4A). The neurons belonging to successful pairs were defined as successful neurons. The other neurons were found to be unsuccessful for either set of inferred antidromic spikes.

##### Evoked spikes

We computed waveform stability and bias of the peak pattern for each evoked spike (Figure 4B). The waveform stability was evaluated on the basis of the Pearson’s correlation of waveforms between the median waveform and the waveform in each no-trigger trial. The waveforms were extracted from −0.25 ms to 0.5 ms around the peak amplitude of spikes. The waveform stability was defined as the median of the Pearson’s correlation coefficients among the stimulation trials. The bias of the peak pattern was calculated as the coefficient of variation among the median peak amplitudes of the four channels of evoked spikes in the no-trigger trials.

##### Relationship of evoked spikes and trigger spikes

We evaluated the similarity of peak patterns, the similarity of waveforms, and the peak amplitudes for each pair of evoked spikes and trigger spikes (Figures 5A and 5B). The similarity of a peak pattern was calculated as the direction cosine used in the online *Multi-Linc* procedure. The similarity of the waveform was calculated as the Pearson’s correlation coefficient of the median waveforms from −0.25 ms to 0.5 ms around the peak amplitude of the spikes. The peak amplitude was extracted from the channel with the maximum peak amplitude of the median waveform among four channels. In addition, we computed the discriminability (i.e., the AUC) between “success” and “other” pairs on the basis of the waveform correlation between the evoked and triggered spikes while varying the width of the waveforms used (Figure 5C). The range of the waveform was varied to 0.5 ms before and after the peak (i.e., the pre-peak range and the post-peak range, respectively).

### Quantification and statistical analysis

#### Statistics

The details of the statistical tests are summarized in Table S2.

## Data Availability

• All data to plot figures in this paper are publicly available in GitHub https://github.com/sakai-tamagawa/MultiLinc. • Original code described in c, c++, perl and shell-script to control the online Multi-Linc controller is publicly available in GitHub https://github.com/sakai-tamagawa/MultiLinc. • Any additional information required to reanalyze the data reported in this paper is available from the lead contact upon request.
