# Supplementary material for: Automated and parallelized spike collision tests to identify spike signal projections
Source: iScience. 2022 Sep 5;25(10):105071. doi: 10.1016/j.isci.2022.105071 (PMC9490030; doi:10.1016/j.isci.2022.105071)
Supplement: Document S1. Figures S1–S6 and Tables S1 and S2 [file mmc1.pdf]

iScience, Volume *25*

Supplemental information

Automated and parallelized spike collision tests to identify spike signal projections

Keita Mitani, Masanori Kawabata, Yoshikazu Isomura, and Yutaka Sakai

Figure S1. Procedure for the offline analysis of the collision test. Related to STAR Methods: “Method details”.

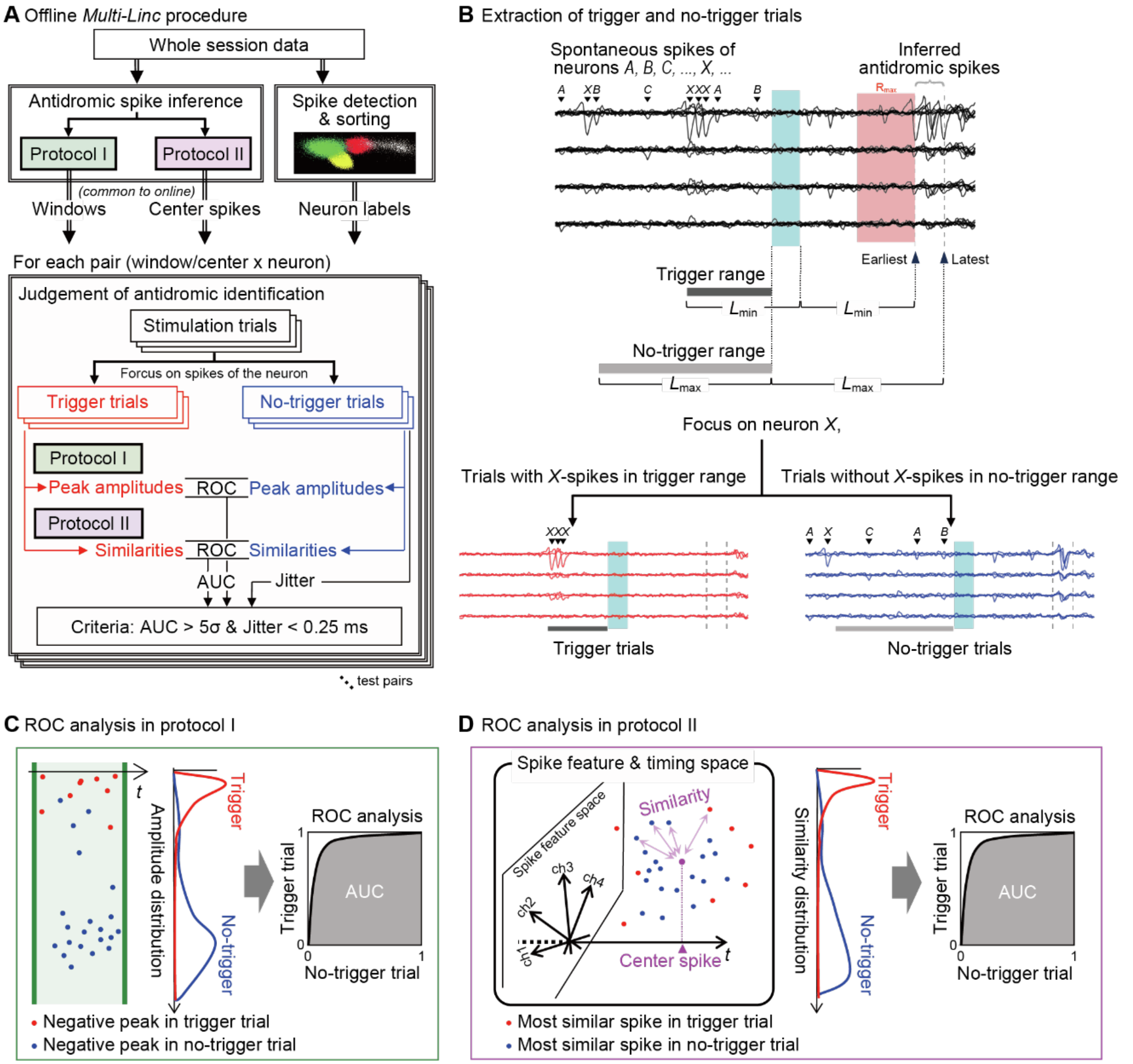

A, Flow of offline *Multi-Linc* procedure. **B**, Procedure for extraction of trigger and no-trigger trials focusing on a certain pair of a neuron and a set of inferred antidromic spikes. To avoid false-positive identification because of neuronal refractory, we excluded trials such that spontaneous spikes occur within R_{max} = 4 ms before the earliest timing of the inferred antidromic spikes (red range). We defined the trigger/no-trigger range (dark-gray/light-gray bar) before the stimulation such that spontaneous spikes in the range *should*/*could* collide with the evoked antidromic spikes. The possible minimum latency is inferred as the length from the offset of the stimulation light to the earliest timing of the inferred antidromic spikes, L_{min}. Hence, a spontaneous spike L_{min} before the light offset

should not yet reach the axon terminal until the light offset and should collide with the evoked antidromic spike. We defined the trigger range from L_{min} before the light offset to the light onset. However, the possible maximum latency is inferred as the length from the light onset to the latest timing of inferred antidromic spikes, L_{max}. Hence, a spontaneous spike L_{max} before the light onset might not yet reach the axon terminal at the light onset and might be in time to collide. We defined the no-trigger range of length L_{max} before the light onset. If spontaneous spikes do not occur in the no-trigger range, then the collision should not occur. In these ranges before optical stimulations, multiple spontaneous spikes of multiple neurons (*A*,*B*,*C*, … *X*, …) occurred. Focusing on a neuron *X*, we extracted the stimulation trials with some spikes of *X* in the trigger range (dark-gray bar) as the trigger trials (red). Conversely, we extracted the trials without any spikes of *X* in the no-trigger range (light-gray bar) as the no-trigger trials (blue). Moreover, the no-trigger trials were restricted so that the terms of the trigger and no-trigger trials in the recording session might be equivalent for the sake of fair comparison (see **STAR Methods**). **C**, **D**, Schema of ROC analyses in protocols I and II to judge whether the pair succeeds in the collision test. Elimination of antidromic spikes inferred to be evoked can be judged on the basis of the variables used in the antidromic spike inference: the negative peak amplitudes in protocol I (**C**) and the similarity to the center spike in protocol II (**D**).

Figure S2. Examples of the offline analysis. Related to STAR Methods: "Method details".

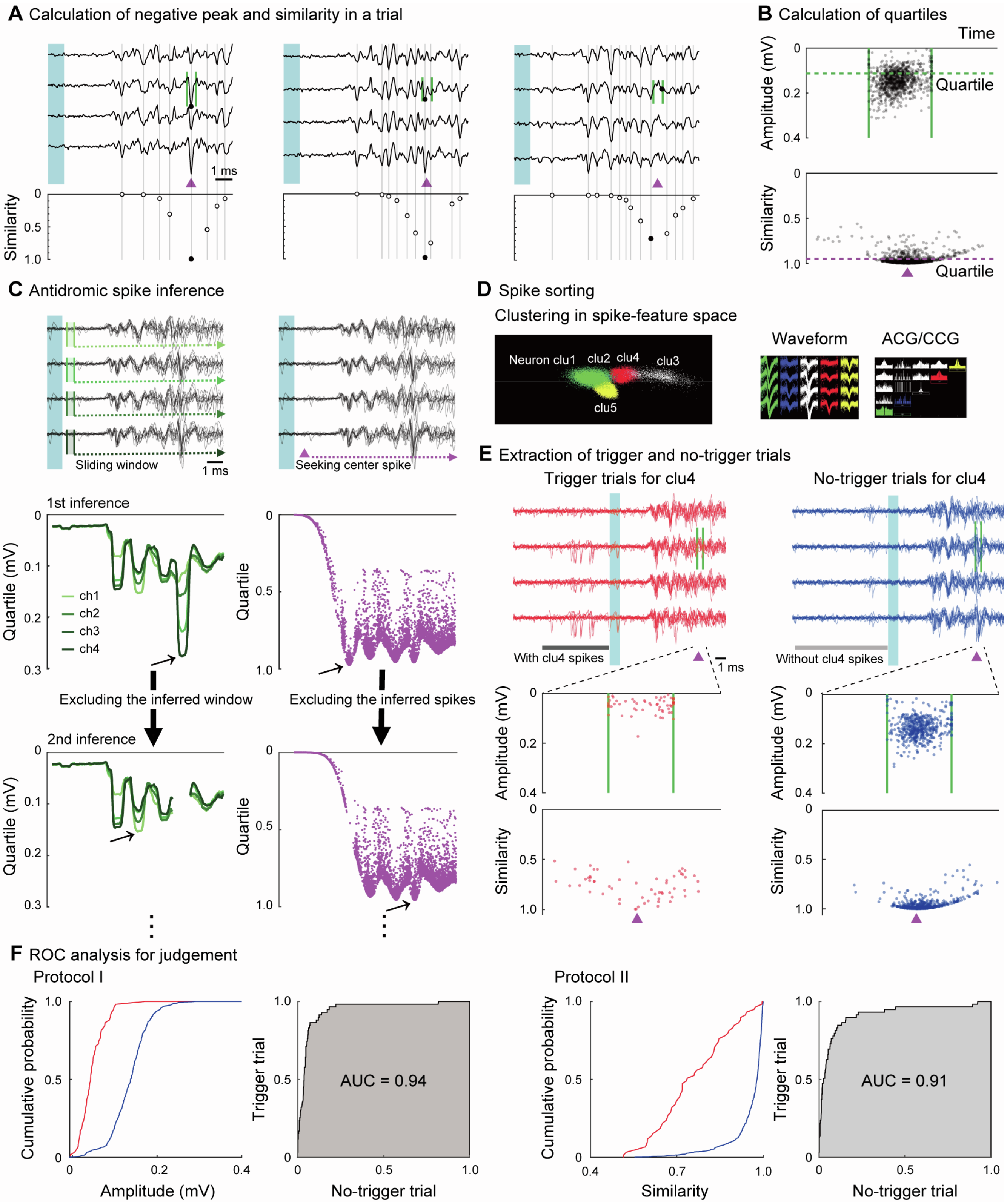

A, Negative peaks (protocol I) and similarities (protocol II) in an example stimulation trial. The negative peaks in a window (green vertical lines) are shown as black dots in each trace. The timing of the focused center spike in a different trial is indicated by a magenta triangle. The similarities in timings and relative peak patterns of evoked spikes in this trial to the center spike are shown in the lower panels. The similarity of the most similar spike to the center spike is adopted in each trial (black filled dot). The highest similarity in a trial gives a high value if an evoked spike exists such that both the timing and relative peak pattern are similar to the center spike (left, center), and gives a low value otherwise (right). **B**, Distributions (scatter dots) and their lower quartiles (75% from the largest; dashed lines) of negative peak amplitudes within a window and highest similarities to a center spike in an example session. Each dot shows the negative peak amplitude (protocol I) and the highest similarity (protocol II) adopted in each trial, plotted against the spike timing. A high quartile value indicates that antidromic-like spikes are stably evoked in stimulation trials. **C**, Examples of antidromic spike inference. Antidromic spike inferences were performed on the basis of the quartiles of peak amplitudes (protocol I) and similarities (protocol II). The quartiles were calculated as functions of all sliding windows (green curves) or all seeking center spikes (magenta dots). The window or the center spike with the maximum quartile (black diagonal arrows) was adopted as the best inference of antidromic spikes. The spikes over the quartile were adopted as a set of inferred antidromic spikes. After the window range or the inferred spikes were excluded, the following window or center spike was searched. **D**, An example of spike sorting. All spontaneous spikes in a session were sorted into clusters likely to originate in identical neurons by a standard spike sorting technique (EToS; see **STAR Methods**) to prepare for extraction of trigger and no-trigger trials. The plots show clusters in spike feature space (left), their overlayed waveforms (center), and ACG/CCG (right). **E**, Extraction of trigger and no-trigger trials for an example pair of a spike cluster (clu4, a putative identical neuron) and an inferred antidromic window (green vertical lines) or center (magenta triangles). For each cluster, trials with spontaneous spikes of the cluster in the trigger range (dark-gray bar; see **Figure S1B**) before stimulation were extracted as the trigger trials (top left, red). Trials without any spontaneous spikes of the cluster in the no-trigger range (light gray bar) were extracted from trials near the trigger trials as no-trigger trials (top right, blue). Negative peak amplitudes within the window (protocol I; middle) and similarities to the center spike (protocol II; bottom) were calculated for the respective sets of trigger (left) and no-trigger (right) trials. The lower value of the peak amplitude or the similarity in each trial indicates that an antidromic spike is more likely to be eliminated or not be evoked. If the peak amplitudes or the similarities in the trigger trials are sufficiently lower than those in the no-trigger trials, then spontaneous spikes of the cluster just before stimulations are likely to collide with the evoked antidromic spikes. **F**, Example of ROC analysis for judgement of whether the spike collision might occur. Cumulative distributions of peak amplitudes (protocol I; left half) or similarities (protocol II; right half) in trigger trials (red) and no-trigger trials (blue) and the ROC curves between trigger and no-trigger trials (black lines) are shown. The ROC curve represents the cumulative fraction at a certain value in the trigger trials as a function of that in the no-trigger trials. A larger area under the ROC curve (AUC; gray area) means a larger segregation between the trigger and no-trigger trials. The success of the collision was determined by a sufficiently large AUC (see **Figure 3C**).

Figure S3. Overlap in identified neuronal projections by protocols I and II. Related to Figure 3.

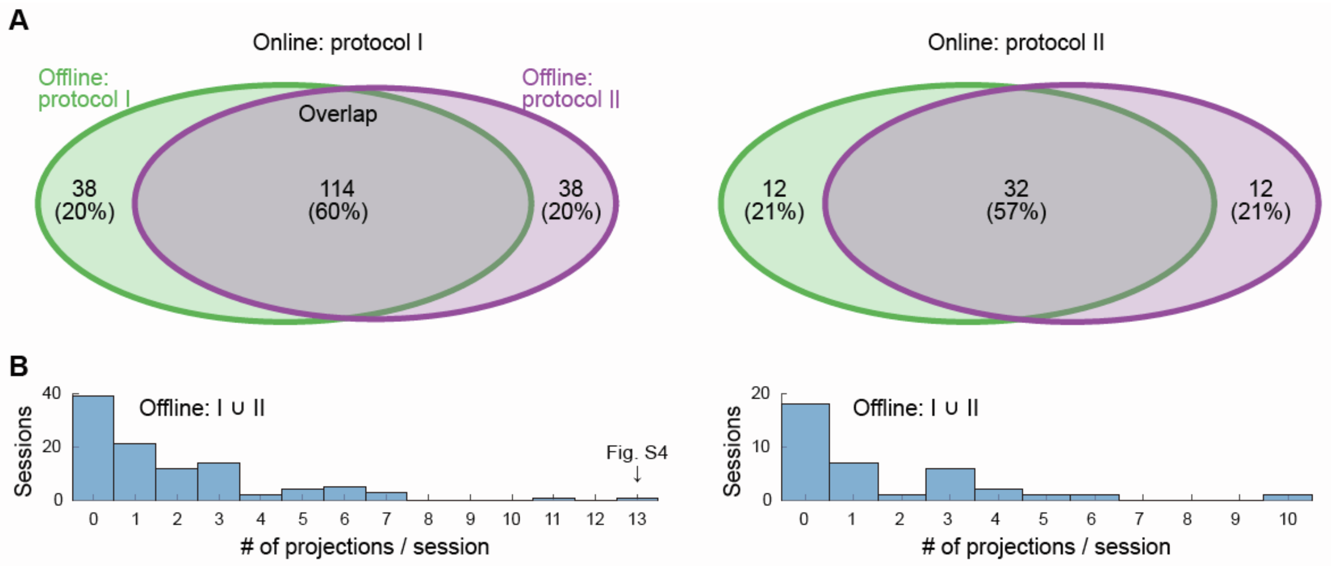

C

	Online: protocol I				Online: protocol II			
Refractory period (ms)	Offline: protocol I	II	I and II	Total	I	II	I and II	Total
1	43	35	124	202	12	13	35	60
2	41	36	124	201	13	10	35	58
3	38	35	121	194	9	11	35	55
4	38	38	114	190	12	12	32	56

A, Venn diagrams of the number of identified neuronal projections in the offline judgements by protocols **I** and **II**, separately shown for the experimental sessions obtained by using the online controllers with protocols I and II (left and right, respectively). The numbers and percentages (parentheses) are described in the respective regions. **B**, Histograms of the merged outcomes of identified neuronal projections per session by protocols **I** and **II**. The maximum merged outcome (13 projections) was obtained in a session using the online controller with protocol I, the details of which are shown in **Figure S4**. **C**, Tables of the number of identified neuronal projections for different presumed refractory periods.

Figure S4. Thirteen successful spike collision tests in a session. Related to Figure 3.

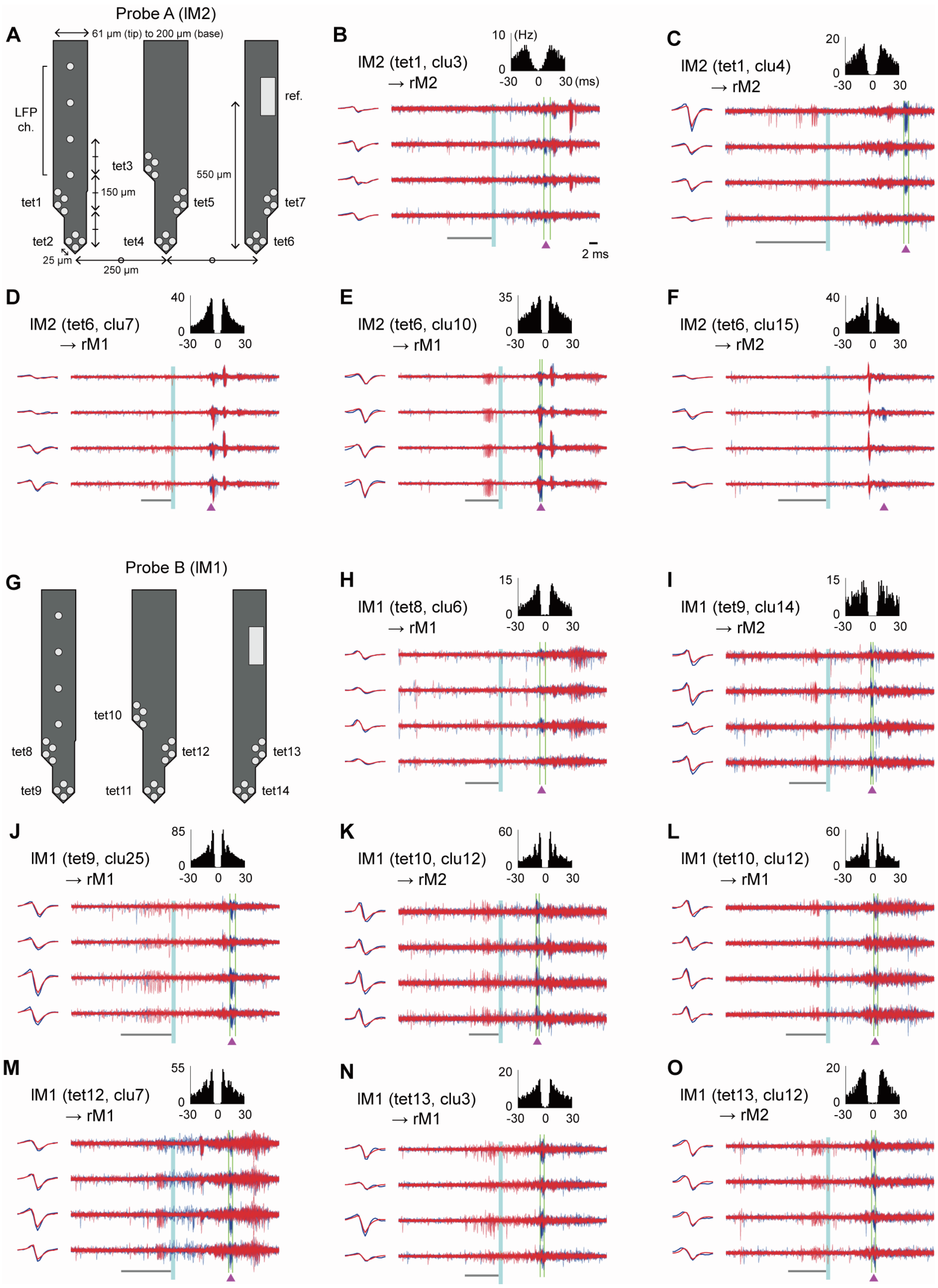

A and **G**, Electrode arrangement of silicone probes A and B. **B–F**, Successful spike collision tests using the *Multi-Linc* system, which simultaneously identified five projection neurons recorded from left M2 (probe A). **H–O**, Successful spike collision tests that identified eight projection neurons in left M1 (probe B). Data are shown in **Fig. 3A**.

Figure S5. Confirmation of the collision judgement in the original scales. Related to Figure 3.

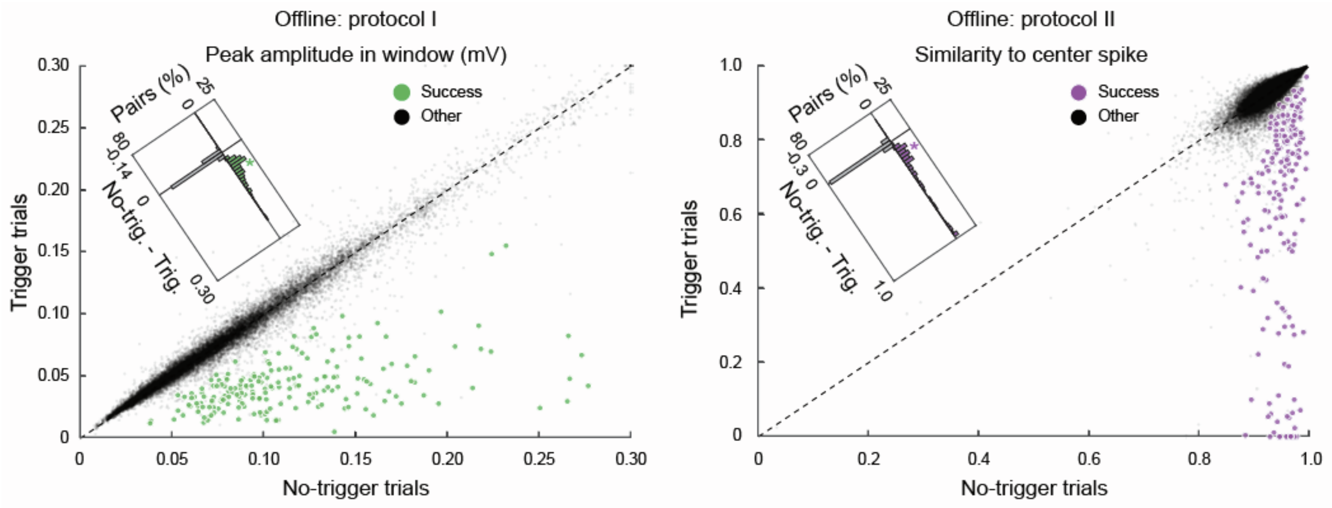

Our judgements of spike collision were based on the nonparametric statistics, AUC of ROC analysis between trigger and no-trigger trials. Here, we confirmed the segregation in the original scales of variables for judgements, the negative peak amplitude in protocol I, and the similarity to the center spike in protocol II. These variables in successfully identified pairs were confirmed to be smaller in trigger trials than in no-trigger trials (green and magenta dots below diagonal dashed lines; $p = 6.5 \times 10^{-34}$ in protocol I, $p = 6.5 \times 10^{-34}$ in protocol II, signed-rank test). The segregations in the successful pairs were larger than the others ($p = 3.4 \times 10^{-138}$ in protocol I, $p = 5.1 \times 10^{-129}$ in protocol II, rank-sum test for differences; diagonal insets).

Figure S6. Other examples of spike collision tests using the *Multi-Linc* system. Related to Figure 3.

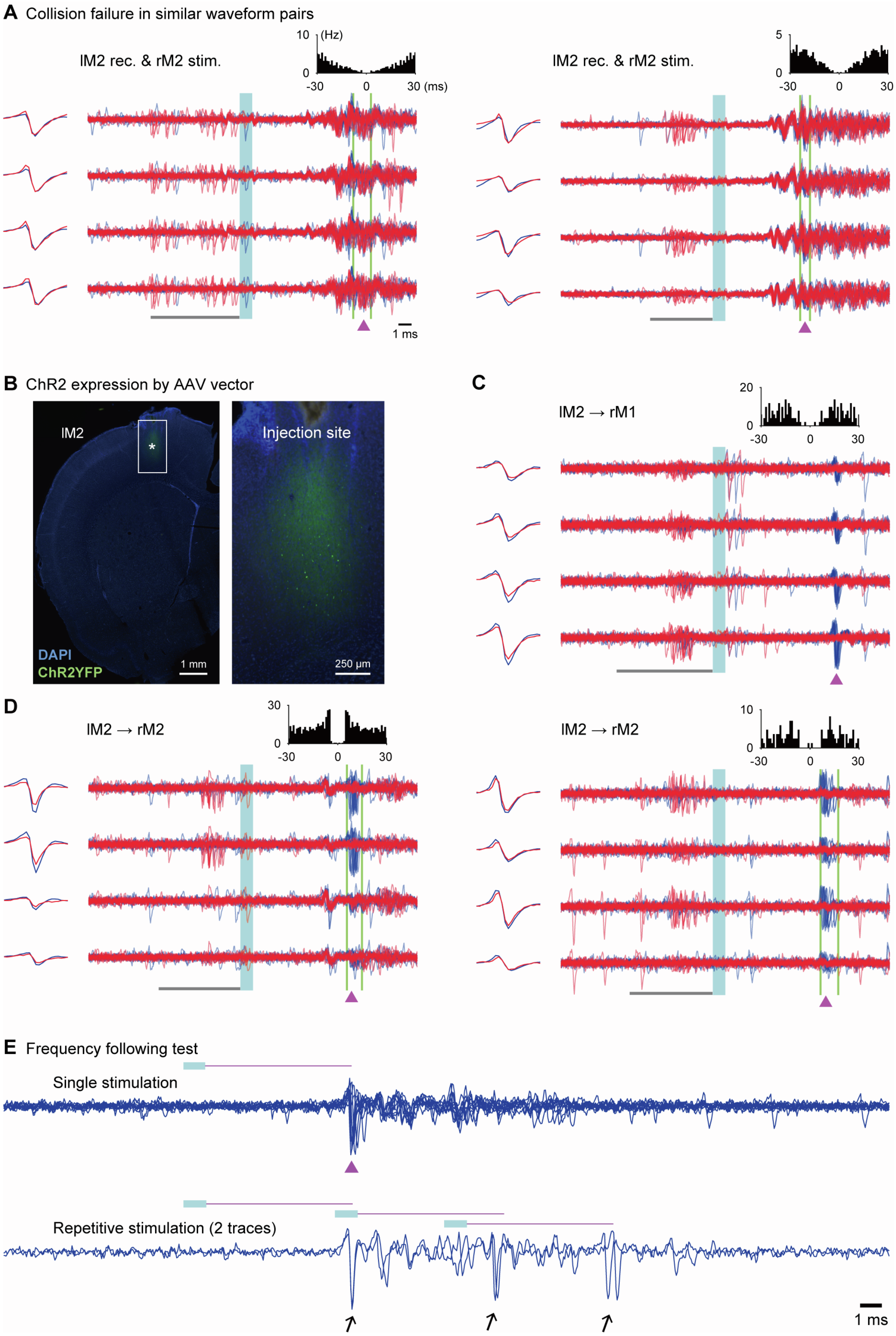

A, Two examples of neurons that did not pass the spike collision test despite their high similarity between trigger and evoked spikes. The waveforms of their trigger spikes were almost identical to those of evoked spikes (>0.9 in Pearson's correlation coefficient), and the jitter of their evoked spikes satisfied the criterion (<0.25 ms). However, the AUC in amplitude/similarity between trigger and no-trigger trials did not satisfy the criterion (<5σ), rejecting their occurrence of spike collision. Data are shown in **Figure 3A**. **B−D**, A preliminary example of a spike collision test in a wild-type (Long–Evans) rat with ChR2 expressed cortically via an AAV vector. **B**, Virally expressed ChR2-EYFP (green; immunostained) in the left M2 (left). An injection site (asterisk in a box) is shown magnified on the right. The *Multi-Linc* experiment was performed six weeks after cortical injection of AAVDJ-Syn-hChR2-EYFP (1.8 × 10^{10} vg/μl, 1 μl/site, provided by Dr. Kenta Kobayashi at the National Institute for Physiological Sciences, Japan) into the left M1 and M2. **C** and **D**, Successful spike collision tests using the *Multi-Linc* system in a session. Data are shown in **Figure 3A**. **E**, Demonstration of a frequency-following test. The *Multi-Linc* system was configured to automatically perform several trials of the frequency-following test for evoked spikes if necessary. Upper, evoked spikes in response to single-pulse stimulation (blue) in a neuron that passed the spike collision test. A magenta triangle with a horizontal line indicates the timing of the center spike and its latency from the stimulation. Lower, similar spikes evoked exactly in response to each pulse of repetitive stimulation (arrows). It satisfied the frequency-following test.

Table S1. Summary of the identified projection neurons. Related to Figure 3.

Online: protocol I ∪ II

Recording site	Stimulation site	Tested probe fiber	Number of projections	Identification rate	Latency (ms)	Jitter (ms)
lM1	lM1	30	1 (1, 0)	3.3%	6.1	0.23
	lM2	6	0	0.0%		
	rM1	168	32 (26, 25)	19.0%	8.0 ± 1.5	0.1 ± 0.04
	rM2	104	7 (6, 6)	6.7%	7.4 ± 2.9	0.08 ± 0.06
lM2	lM1	38	9 (9, 6)	23.7%	6.2 ± 2.8	0.08 ± 0.06
	lM2	34	26 (19, 19)	76.5%	5.1 ± 1.2	0.07 ± 0.04
	rM1	162	23 (17, 18)	14.2%	8.9 ± 2.2	0.08 ± 0.03
	rM2	210	137 (109, 116)	65.2%	9.9 ± 2.4	0.11 ± 0.04
rM1	lM1	18	1 (1, 1)	5.6%	19.8	0.13
	lM2	2	0	0.0%		
	rM1	18	1 (1, 1)	5.6%	6.6	0.18
	rM2	2	0	0.0%		
rM2	lM1	2	0	0.0%		
	lM2	30	4 (3, 2)	13.3%	10.8 ± 0.7	0.18 ± 0.02
	rM1	2	0	0.0%		
	rM2	34	5 (4, 2)	14.7%	4.5 ± 1.6	0.09 ± 0.05

For each pair of a recording site and a stimulation site, we summarized the number of tested pairs of probes and fibers, the number of identified projection neurons, the identification rate of projection neurons to the tested pairs, the latency of evoked spikes (median ± quartile deviation), and the jitter of those (median ± quartile deviation) for online protocols I and II. The numbers in parentheses in "Number of projections" indicate those for offline judgements by protocols I and II, respectively. The latency and jitter were shorter for ipsilateral projections than for contralateral ones ($p = 3.3 \times 10^{-9}$ and $p = 0.0081$, respectively, rank-sum test).

Table S2. Summary of the statistical parameters. Related to Figures 3−5.

Figure	Test	Protocol	Descriptive statistics	DF	Test statistic	Effect size
Fig. 3C	χ^2 test	I	OR = 0.10 (0.07, 0.14)	1	χ^2 = 248.34	φ = 0.07
		II	OR = 0.22 (0.15, 0.33)	1	χ^2 =67.93	φ = 0.03
Fig. 3D	Pearson's correlation	I	r = 0.48 (0.35, 0.58)	194	Same as left	n/a
		II	r = 0.53 (0.41, 0.62)	194		
Fig. 4A	χ^2 test	I	OR = 0.13 (0.05, 0.36)<br>Narrow: 0.19%, Wide: 1.42%	1	χ^2 = 22.13	φ = 0.04
		II	OR = 0.03 (0.00, 0.22)<br>Narrow: 0.04%, Wide: 1.53%	1	χ^2 = 30.30	φ = 0.05
Fig. 4B	Rank-sum test (waveform stability)	I	Success: M = 0.86 (0.84, 0.87)<br>Other: M = 0.75 (0.74, 0.75)	5062	z = 12.73	δ = 0.54
		II	Success: M = 0.88 (0.87, 0.89)<br>Other: M = 0.69 (0.69, 0.70)	7999	z = 16.43	δ = 0.73
	Rank-sum test (bias of peak-pattern)	I	Success: M= 0.38 (0.34, 0.42)<br>Other: M = 0.22 (0.21, 0.22)	5062	z = 11.04	δ = 0.47
		II	Success: M = 0.40 (0.36, 0.44)<br>Other: M = 0.13 (0.13, 0.14)	7999	z = 15.60	δ = 0.70
Fig. 5A	Rank-sum test (similarity of waveform)	I	Success: M= 0.97 (0.97, 0.98)<br>Other: M = 0.77 (0.77, 0.78)	19083	z = 22.15	δ = 0.92
		II	Success: M = 0.97 (0.97, 0.98)<br>Other: M = 0.78 (0.78, 0.78)	34161	z = 21.11	δ = 0.87
Fig. 5B	Linear regression	I	a_0 = 0.004 (–0.004, 0.012)	194	t = 1.01	R^2 = 0.75
			a_1 = 0.74 (0.68, 0.80)	194	t = 24.41	
		II	a_0 = 0.010 (0.002, 0.017)	194	t = 2.59	R^2 = 0.75
			a_1 = 0.70 (0.64, 0.75)	194	t = 24.36	
	Signed-rank test (evoked vs. trigger)	I	M = 0.02 (0.02, 0.03)	195	z = 11.49	r = 0.82
		II	M = 0.02 (0.02, 0.02)	195	z = 11.51	r = 0.82

Continue to the next page

Figure	Test	Protocol	Descriptive statistics	DF	Test statistic	Effect size
Fig. S5	Signed-rank test (no-trigger vs. trigger)	I	M = 0.06 (0.05, 0.07)	195	z = 12.14	r = 0.87
		II	M = 0.21 (0.19, 0.25)	195	z = 12.14	r = 0.87
	Rank-sum test	I	Success: M = 0.060 (0.050, 0.070)<br>Other: M = 1.0×10^{-4} (5.0×10^{-5}, 1.5×10^{-4})	19083	z = 25.02	δ = 0.995
		II	Success: M = 0.21 (0.19, 0.25)<br>Other: M = 6.4×10^{-4} (5.3×10^{-4}, 7.5×10^{-4})	34161	z = 24.54	δ = 0.993

For each figure, we summarized the type of test, offline protocol, descriptive statistics, degree of freedom (DF), test statistic, and effect size. All tests were two-tailed. For descriptive statistics, OR, M, and a_k are odds ratio, median, and *k*-th regression coefficient, respectively. The numbers in parentheses in “Descriptive statistics” indicate 95% confidence intervals calculated by bootstrap. Their *p*-values are given in the main text or this supplementary information. The success rates of collision tests for narrower and wider waveform groups are shown for **Figure 4A**.
